# Understanding spatiotemporal clustering of seasonal influenza in the United States

**DOI:** 10.1186/s12879-026-13000-7

**Published:** 2026-03-04

**Authors:** Louis Yat Hin Chan, Sinead Morris, Norman Hassell, Perrine Marcenac, Alexia Couture, Arielle Colon, Krista Kniss, Alicia Budd, Matthew Biggerstaff, Rebecca Borchering

**Affiliations:** 1 Influenza Division, Centers for Disease Control and Prevention, 1600 Clifton Road, Atlanta, GA 30329 USA; 2 Goldbelt Professional Services, Chesapeake, VA USA

**Keywords:** Time series clustering, Moran’s I statistic, Spatiotemporal heterogeneity, Disease surveillance, Respiratory diseases, Influenza

## Abstract

**Background:**

Seasonal influenza exhibits distinct spatiotemporal patterns across the United States, yet the geographic clustering of influenza activity remains incompletely understood. This study aims to identify jurisdictions with similar patterns of seasonal influenza epidemics by exploring spatiotemporal dynamics across the United States after the 2009 H1N1 pandemic.

**Methods:**

We analyzed data from U.S. influenza surveillance systems, including outpatient illness surveillance and virologic surveillance. The outpatient illness data included weekly proportions of outpatient visits for influenza-like illness from jurisdictions including all 50 states, while virologic data comprised influenza test positivity results from U.S. public health and clinical laboratories covering all 50 states. We calculated Moran’s I statistics to assess spatial autocorrelation in peak timing. We also performed k-means clustering on z-normalized time series data and determined optimal clusters using the silhouette method. We then conducted an analysis of variance (ANOVA) to evaluate differences among clusters based on the Moran’s I statistics and the relative proportions of influenza virus types and subtypes.

**Results:**

Our analysis revealed distinct spatial clusters with significant geographic patterns. We found a consistent grouping of Southeastern states (Georgia, Alabama, Mississippi, Louisiana, and Florida). This clustering pattern was partially explained by earlier seasonal peaks in these jurisdictions and supported by significant spatial autocorrelation in peak timing. While Southeastern states maintained stable cluster associations, Western and Central states showed greater variation in cluster membership across seasons. We also found significant differences between clusters in the Moran’s I statistics and the proportion of all influenza A virus detections that were influenza A/H1 viruses. However, no significant differences were found in the proportion of all influenza A and B virus detections that were influenza A viruses.

**Conclusions:**

These findings quantify the distinct spatiotemporal patterns of seasonal influenza in the Southeastern United States compared to other regions, and highlight a consistent cluster characterized by earlier epidemic timing across seasons and surveillance indicators. Understanding these regional clustering patterns can enhance preparations for upcoming changes in influenza activity and inform targeted public health interventions such as timing of vaccination campaigns and regional situational awareness. Robust surveillance systems, adaptive approaches, and stable long-term data are essential for effectively addressing regional differences and ultimately strengthening nationwide preparedness for seasonal influenza.

**Supplementary Information:**

The online version contains supplementary material available at 10.1186/s12879-026-13000-7.

## Introduction

Seasonal influenza remains a major public health concern worldwide, characterized by substantial morbidity, mortality, and economic impacts [1–5]. Influenza viruses are primarily classified into influenza types A and B, with influenza A viruses (subtypes A/H1 and A/H3) generally causing the majority of infections [6]. The burden of seasonal influenza in the United States is significant and varies by season [7]. For example, the 2017/2018 influenza season alone resulted in an estimated 35–52 million symptomatic illnesses, 550,000–1 million hospitalizations, and 36,000–98,000 deaths [8].

Influenza outbreaks exhibit marked temporal patterns, with activity typically beginning to increase in November and peaking between December and February in the United States [9]. While these temporal dynamics have been well-studied [10], revealing consistent seasonality across years, there remains a significant research gap in whether seasonal influenza epidemics vary by geography within the United States. Surveillance data indicate that seasonal influenza epidemics in the United States frequently, but not always, originate in the Southeastern region, particularly in states such as Georgia, before spreading to other areas [11]. However, these spatial patterns are dynamic and vary annually due to complex interactions between climate, population density, human mobility, and viral evolution [12–17]. This variability complicates efforts to predict where influenza activity will increase next and presents challenges for effective preparedness and response to seasonal influenza outbreaks.

Several studies have contributed to our understanding of influenza spatial dynamics in the United States. Gog et al. [18] and Kissler et al. [19] demonstrated that the fall wave of the 2009 influenza pandemic activity originated in the Southeastern United States, while Charu et al. [12] identified clear spatial patterns in influenza onset times across the United States from 2002 to 2010, with most seasons starting in the Southern United States. Rosensteel et al. [20] further highlighted significant heterogeneity in regional clustering patterns across the seasons from 2002 to 2009. Although Dahlgren et al. [21] found no evidence of meaningful spatial autocorrelation in peak timing for the 2010–2016 seasons, Thivierge et al. [22] showed that incorporating spatial information into influenza forecasting models using U.S. surveillance data from 2010 to 2019 improved predictive accuracy, highlighting the continued relevance of spatial structure. Despite these insights, there remains limited understanding of how spatial patterns have evolved over time, particularly in the years following the 2009 H1N1 pandemic.

Spatial clustering refers to the grouping of regions with similar patterns of influenza activity, which can provide insights into the spread of influenza within a country. By examining these spatiotemporal dynamics, we can enhance our understanding of influenza spread and the variability in regional activity. This study examines the spatial clustering of influenza activity across the United States after the 2009 H1N1 pandemic. We first analyze spatiotemporal dynamics by examining the peak timing of time series data from influenza outpatient illness surveillance and from virologic surveillance systems [23]. The former captures visits for influenza-like illness (ILI), defined as fever with cough or sore throat, which correlates strongly with influenza virus activity, whereas the latter is based on laboratory-confirmed detections of influenza virus. Then, we identify spatial clusters by applying k-means clustering, an unsupervised machine learning technique, to the time series data. Finally, we apply analysis of variance (ANOVA) to explain the regional differences in influenza dynamics. Our findings contribute to the broader field of spatiotemporal epidemiology and offer insights that can help inform prevention and control efforts for future influenza seasons.

## Methods

### Surveillance data and preprocessing

This study utilized two primary surveillance systems: outpatient illness surveillance and virologic surveillance. First, we used unweighted weekly proportions of outpatient visits for ILI from the U.S. Outpatient Influenza-like Illness Surveillance Network (ILINet). These data may capture a range of respiratory infections caused by pathogens other than influenza viruses. Second, we used weekly values of the percentage of specimens testing positive for influenza virus from virologic surveillance, which includes data from the U.S. World Health Organization (WHO) Collaborating Laboratories System and the National Respiratory and Enteric Virus Surveillance System (NREVSS). These data included test results from approximately 100 public health laboratories, which test respiratory specimens for surveillance purposes, and 300 clinical laboratories, which focus on diagnostic testing. For the period before the 2015/2016 season, both public health and clinical laboratory data were reported together. However, from the 2015/2016 season onward, they were reported separately [23], with weekly values at the state and territory level available only for the clinical laboratory data. Both surveillance systems are voluntary networks with incomplete and variable coverage across jurisdictions and seasons, and the reported data should be interpreted as surveillance indicators rather than direct measures of population incidence [23].

All data from the 2010/2011 to 2023/2024 seasons were sourced from FluView Interactive [23], with the weekly data collected by epidemiologic week, referred to as MMWR Week [24]. In this study, each season was defined as beginning in Week 40 (around early October) each year and ending in Week 39 the following year. The study period focused on two phases: the pre-COVID-19 period (2010/2011–2019/2020) and the post-COVID-19 period (2022/2023–2023/2024). All 50 states, Puerto Rico, and the District of Columbia were covered in both surveillance systems. The outpatient illness surveillance also included the U.S. Virgin Islands and New York City, for a total of 54 jurisdictions. New York City and New York state were reported as separate jurisdictions and treated as distinct entities for the purposes of this study. As a result, the U.S. Virgin Islands and New York City were excluded from analyses using the virologic surveillance data. Data from the territories of American Samoa, Guam, and the Northern Mariana Islands were not available in either surveillance system.

Missing weekly values (the unweighted weekly proportions of outpatient visits for ILI and the weekly percentages of specimens testing positive for influenza) occurred in both datasets, with the latter particularly affected during periods outside the core influenza season due to lower testing volume and variability in voluntary reporting across jurisdictions (Figure S1, Figure S2 and Figure S3). For example, the weekly percentages of specimens testing positive for influenza were reported only during the core influenza period or selected seasons in some jurisdictions. We imputed the missing weekly values for each jurisdiction using Stineman interpolation, implemented with the `imputeTS` package in R [25]. Jurisdictions with more than 50% missing weekly values within a season were excluded from that season in our analyses.

To reduce noise and elucidate the temporal patterns in the data, we applied smoothing using Nadaraya–Watson kernel regression with a Gaussian kernel and a bandwidth of 4 weeks [26]. This step ensured that seasonal trends were more discernible by minimizing short-term fluctuations. Additional details on the smoothing procedure are provided in the Supplementary Material.

To analyze trends over a period of $T$ seasons, we then averaged the smoothed data across seasons for each jurisdiction as follows

$${\stackrel{-}{y}}_{i}=\frac{1}{T}{\sum}_{j=1}^{T}{y}_{ij,}$$

where ${\stackrel{-}{y}}_{i}$ is the average value for week $i$, and ${y}_{ij}$ is the value for week $i$ in season $j$. We note that data for week 53 were excluded from the averaged dataset but were kept when analyzing individual seasons. The weekly data (for individual seasons and averaged across seasons) were used in subsequent peak timing spatial analysis and time series clustering analysis. We note that peak timing estimated from the averaged datasets represents a summarized seasonal profile and is not equivalent to averaging peak timing identified from individual seasons. For the time series clustering analysis, because the two surveillance indicators reflect different surveillance processes and epidemiologic meanings, analyses were conducted separately for each dataset, as well as jointly to assess shared spatiotemporal patterns.

Finally, the public health laboratory data from the virologic surveillance system provided raw detection counts for each influenza virus type (A and B) and subtype (A/H1 and A/H3) by season and jurisdiction. We calculated the seasonal proportions of each type and subtype and included these as dependent variables when exploring cluster differences in a subsequent ANOVA.

### Peak timing spatial analysis

We calculated global and local Moran’s I statistics for each dataset (the unweighted weekly proportions of outpatient visits for ILI and the weekly percentages of specimens testing positive for influenza) to investigate the spatial autocorrelation in timing of peak influenza activity. Peak timing reflects when influenza activity reaches its maximum within a season and provides a concise summary of relative epidemic timing across jurisdictions. Moran’s I statistics evaluate the degree to which the values in each jurisdiction are similar to those in neighboring jurisdictions, providing a measure of spatial clustering. For this analysis, only continental U.S. jurisdictions (including New York City) were included, while jurisdictions such as Alaska, Hawaii, Puerto Rico, and the U.S. Virgin Islands were excluded.

To measure spatial autocorrelation across the United States, we calculated the global Moran’s I statistic and its associated p-value using a permutation test implemented through the `spdep` package in R [27]. Positive values indicate positive spatial autocorrelation or clustering, where jurisdictions with similar peak timing are geographically close to one another. Values near zero suggest a random spatial distribution with no discernible pattern, while negative values indicate negative spatial autocorrelation or dispersion, where jurisdictions with dissimilar peak timing are more likely to be neighbors. The formula for the global Moran’s I is

$$I=\frac{N}{{\sum}_{i=1}^{N}{\sum}_{j=1}^{N}{w}_{ij}}\frac{{\sum}_{i=1}^{N}{\sum}_{j=1}^{N}{w}_{ij}({x}_{i}-\stackrel{-}{x})({x}_{j}-\stackrel{-}{x})}{{\sum}_{i=1}^{N}{({x}_{i}-\stackrel{-}{x})}^{2}},$$

where $N$ is the total number of jurisdictions, ${w}_{ij}$ represents the spatial weight between jurisdictions $i$ and $j$, ${x}_{i}$ is the peak timing at jurisdiction $i$ and $\stackrel{-}{x}$ is the average peak timing across all jurisdictions. We define the spatial weights using a binary adjacency structure, where neighboring jurisdictions are assigned a weight of 1 and non-neighboring jurisdictions a weight of 0. The resulting weight matrix is then row-standardized such that weights for each jurisdiction sum to one.

In addition to the global Moran’s I, we calculated local Moran’s I for each jurisdiction to identify localized patterns of spatial autocorrelation, such as differences within specific clusters or the presence of outliers. The local Moran’s I was also computed using the `spdep` package in R [27]. The formula for the local Moran’s I is

$${I}_{i}=\frac{(N-1)({x}_{i}-\stackrel{-}{x})}{{\sum}_{i=1}^{N}{\left({x}_{i}-\stackrel{-}{x}\right)}^{2}}{\sum}_{j=1}^{N}{w}_{ij}({x}_{j}-\stackrel{-}{x}).$$

### Time series clustering analysis

We performed a k-means clustering analysis using the `dtwclust` package in R [28]. The analysis aimed to group jurisdictions with similar influenza activity patterns over time, ensuring that time series within the same cluster followed comparable temporal trends while remaining distinct from those in other clusters. To quantify differences in temporal trends, we used the Manhattan L1 distance measure.

To ensure comparability across jurisdictions and seasons, we first applied z-normalization to standardize the smoothed time series data and place all data on a consistent scale. For k-means clustering, we then used the silhouette method [29] to determine the number of clusters ($k$) that maximizes the separation between clusters while minimizing the variance within clusters. We explored a range of potential cluster counts up to a maximum of $k=10$. We calculated the silhouette score for each value of $k$ and selected the $k$ with the highest silhouette score.

To ensure robustness, we repeated the clustering 100 times with different random initializations (i.e., different random seeds) and selected the solution with the highest consistency across these runs. Clusters containing only a single jurisdiction were manually excluded from the analysis, while the remaining multi-jurisdiction clusters were retained. These single-jurisdiction clusters occurred infrequently and reflected jurisdiction-specific temporal patterns rather than shared regional structure. Their exclusion was intended to avoid over-interpretation of isolated patterns that do not represent meaningful spatial groupings. We also examined the dispersion of jurisdictions within each cluster, calculating the average intra-cluster Manhattan L1 distance in terms of standard deviations, to identify outliers and assess the clustering quality. This metric helped assess whether jurisdictions within the same cluster were closely grouped or exhibited substantial variability.

We conducted the above clustering analysis by first stratifying each dataset (the unweighted weekly proportions of outpatient visits for ILI and the weekly percentages of specimens testing positive for influenza) separately in univariate analyses. Then, we assessed joint clustering patterns by performing a multivariate analysis that combined both datasets. Note that in the multivariate analysis, a jurisdiction had to be available in both datasets in order to be included (e.g., New York City was excluded because the weekly percentages of specimens testing positive for influenza were not available).

### Evaluating cluster differences using ANOVA

Following the peak timing spatial analysis and time series clustering analysis, we conducted an ANOVA to assess differences among clusters based on four dependent variables: peak timing, local Moran’s I, the proportion of all influenza A and B virus detections that were influenza A viruses, and the proportion of all influenza A virus detections that were influenza A/H1 viruses. This analysis was performed on clustering patterns across individual seasons (not averaged across seasons) to capture season-to-season variations, and we focused on the pre-COVID-19 era (2010/2011–2019/2020) to exclude differences associated with pandemic-related disruptions. The model included three effects: cluster, season, and dataset, with the interaction between these effects also considered. The model was specified as

$$Z={\beta}_{C}\mathrm{C}+{\beta}_{S}\mathrm{S}+{\beta}_{D}\mathrm{D}+{\beta}_{C:S}(\mathrm{C}\times\mathrm{S})+{\beta}_{C:D}(\mathrm{C}\times\mathrm{D})+{\beta}_{S:D}(S \times D)+{\beta}_{C:S:D}(\mathrm{C}\times\mathrm{S}\times\mathrm{D})$$

where $Z$ is the dependent variable, $\mathrm{C}$, $\mathrm{S}$, and $\mathrm{D}$ represent the effects of cluster, season, and dataset, respectively, and ${\beta}_{\bullet}$ represent the model coefficients. To account for multiple comparisons [30], we then performed a post hoc analysis using the Tukey’s honestly significant difference (Tukey HSD) test [31]. Further details are provided in the supplementary materials.

## Results

### Preprocessing surveillance data

We included data from all 54 jurisdictions for most seasons in the unweighted weekly proportions of outpatient visits for ILI from the outpatient illness surveillance, with two exceptions: data from the U.S. Virgin Islands (VI) were excluded for the 2010/2011 season, and data from Puerto Rico (PR) were excluded for three seasons (2010/2011–2012/2013). For the weekly percentages of specimens testing positive for influenza from the virologic surveillance, fewer jurisdictions were included (ranging from 40 to 47 out of 54), as several jurisdictions had more than 50% missing weekly values in some seasons (Figure S1, Figure S2 and Figure S3). All seasons were excluded for the District of Columbia (DC), New Jersey (NJ) and Rhode Island (RI) due to insufficient data. Approximately 6% of the weekly values were imputed in the included jurisdictions (Table S1).

### The Southeastern states experienced earlier peak timing

**Fig. 1:**
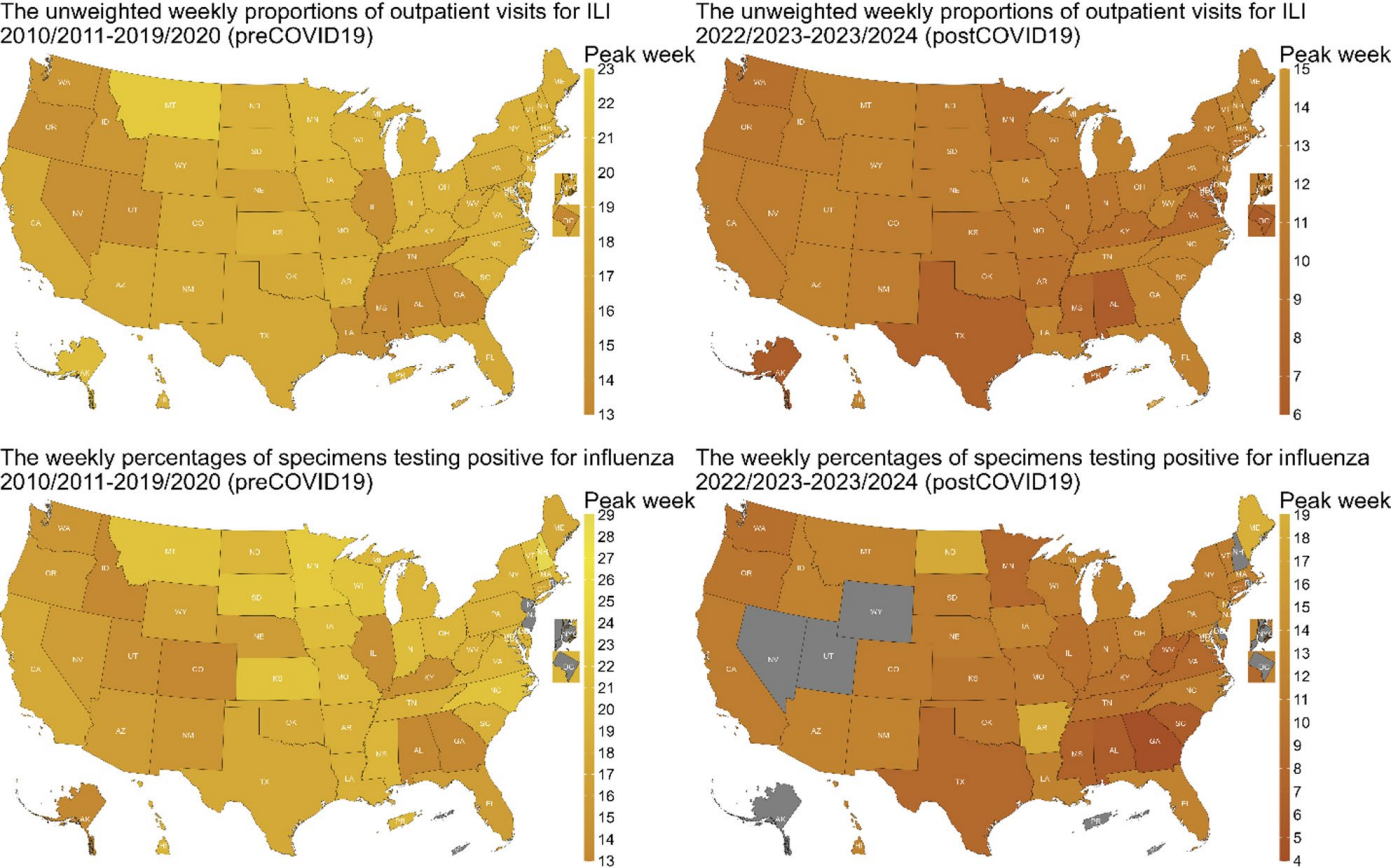
Spatial distribution of peak timing for the unweighted weekly proportions of outpatient visits for ILI (upper panels) and the weekly percentages of specimens testing positive for influenza (lower panels), averaged across seasons. The latter consist of both public health and clinical laboratory data reported before the 2015/2016 season, while only the clinical laboratory data were available from the 2015/2016 season onward. The left panels represent the pre-COVID-19 seasons (2010/2011–2019/2020), while the right panels represent the post-COVID-19 seasons (2022/2023–2023/2024). Darker yellow indicates earlier peak timing, while lighter yellow represents later peak timing. All panels share the same color scale, which corresponds to the week from the beginning of influenza season (MMWR Week 40). The gray color (e.g., in DC, NJ, and NYC) indicates that all seasons were excluded from the analysis due to missing data

Our analysis revealed that peak timing, based on the averaged time series data for the pre-COVID-19 period (2010/2011–2019/2020), occurred earlier in the Southeastern states for both datasets (the unweighted weekly proportions of outpatient visits for ILI and the weekly percentages of specimens testing positive for influenza) (Fig. 1). Specifically, Georgia (GA) and Alabama (AL) exhibited peak timing approximately 5 weeks earlier than the national average across all jurisdictions, based on the averaged time series. These differences indicate systematic variation in the timing of epidemic progression across regions. Additionally, some Western states also exhibited earlier peak timing. For example, Utah (UT) peaked approximately 4 weeks earlier. In contrast, Montana (MT) experienced peak timing about 4 to 5 weeks later than the average. While both datasets demonstrated similar spatial patterns, slight variations were observed between them. For example, the weekly percentages of specimens testing positive for influenza virus showed less consistency in peak timing compared to the unweighted weekly proportions of outpatient visits for ILI, with a broader range of peak weeks across states, particularly in the Central and Northeastern states. For the post-COVID-19 period (2022/2023–2023/2024), the peaks generally occurred earlier than in the pre-COVID-19 period. In addition, we conducted supplementary analyses comparing peak timing estimated from the averaged datasets with the median peak timing derived from individual seasons (Figure S4, Figure S5, and Figure S6).

For the pre-COVID-19 period, the global Moran’s I values were 0.29 for the unweighted weekly proportions of outpatient visits for ILI and 0.30 for the weekly percentages of specimens testing positive for influenza, with both p-values < 0.05, indicating statistically significant spatial autocorrelation (Table 1). These results suggest that jurisdictions with similar peak timings tend to cluster geographically. Further analysis using the local Moran’s I revealed distinct localized spatial patterns between the two datasets (Figure S7). For the unweighted weekly proportions of outpatient visits for ILI, higher local Moran’s I values were observed in Southeastern states such as Alabama (AL), while for the weekly percentages of specimens testing positive for influenza, higher values were found in Western states such as Utah (UT). These findings highlight specific regions where clustering of peak timing is stronger and demonstrate that spatial associations varied across jurisdictions and datasets. In contrast, our analysis for the post-COVID-19 period revealed distinct differences compared to the pre-COVID-19 period. The global Moran’s I values were lower for both datasets, indicating weaker spatial autocorrelation (Table 1).

We also examined peak timing during individual seasons (Figure S8 and Figure S9) to characterize inter-seasonal variability. We found that the global Moran’s I values varied across seasons (Table 1), with more than half showing statistically significant spatial autocorrelation (p-values < 0.05). This suggests that while spatial clustering of peak timing is a consistent feature overall, its strength fluctuates from season to season. In particular, the global Moran’s I values were not significant for the 2020/2021 season, due to notably lower influenza activity during the COVID-19 pandemic. However, the 2022/2023 and 2023/2024 seasons showed higher global Moran’s I values with p-values < 0.05, indicating the re-emergence of spatial clustering and a gradual return to more typical spatial patterns of influenza activity. Similarly, the local Moran’s I values also exhibited seasonal variability, reflecting changes in the strength and patterns of localized spatial clustering (Figure S10 and Figure S11).

**Table 1:** Global Moran’s I values (with p-values in parentheses) of peak timing for the unweighted weekly proportions of outpatient visits for ILI and the weekly percentages of specimens testing positive for influenza, across multiple seasons and single seasons. The latter consist of both public health and clinical laboratory data reported before the 2015/2016 season, while only the clinical laboratory data were available from the 2015/2016 season onward. Bold values indicate statistically significant spatial autocorrelation (*p* < 0.05)

Study period	The unweighted weekly proportions of outpatient visits for ILI	The weekly percentages of specimens testing positive for influenza
**Multiple seasons**		
2010/2011–2019/2020	**0.29 (< 0.05)**	**0.30 (< 0.05)**
2022/2023–2023/2024	**0.20 (< 0.05)**	0.04 (0.25)
**Single season**		
2010/2011	**0.27 (< 0.05)**	**0.38 (< 0.05)**
2011/2012	0.00 (0.39)	0.08 (0.13)
2012/2013	**0.45 (< 0.05)**	-0.03 (0.35)
2013/2014	**0.36 (< 0.05)**	**0.13 (< 0.05)**
2014/2015	**0.60 (< 0.05)**	-0.04 (0.56)
2015/2016	0.08 (0.15)	0.11 (0.11)
2016/2017	**0.49 (< 0.05)**	**0.17 (< 0.05)**
2017/2018	**0.42 (< 0.05)**	**0.25 (< 0.05)**
2018/2019	**0.18 (< 0.05)**	**0.28 (< 0.05)**
2019/2020	-0.01 (0.43)	**0.19 (< 0.05)**
2020/2021	-0.08 (0.71)	0.02 (0.31)
2021/2022	0.05 (0.19)	**0.25 (< 0.05)**
2022/2023	**0.60 (< 0.05)**	**0.34 (< 0.05)**
2023/2024	**0.20 (< 0.05)**	**0.21 (< 0.05)**

### Consistent clustering patterns in the Southeastern states

**Fig. 2:**
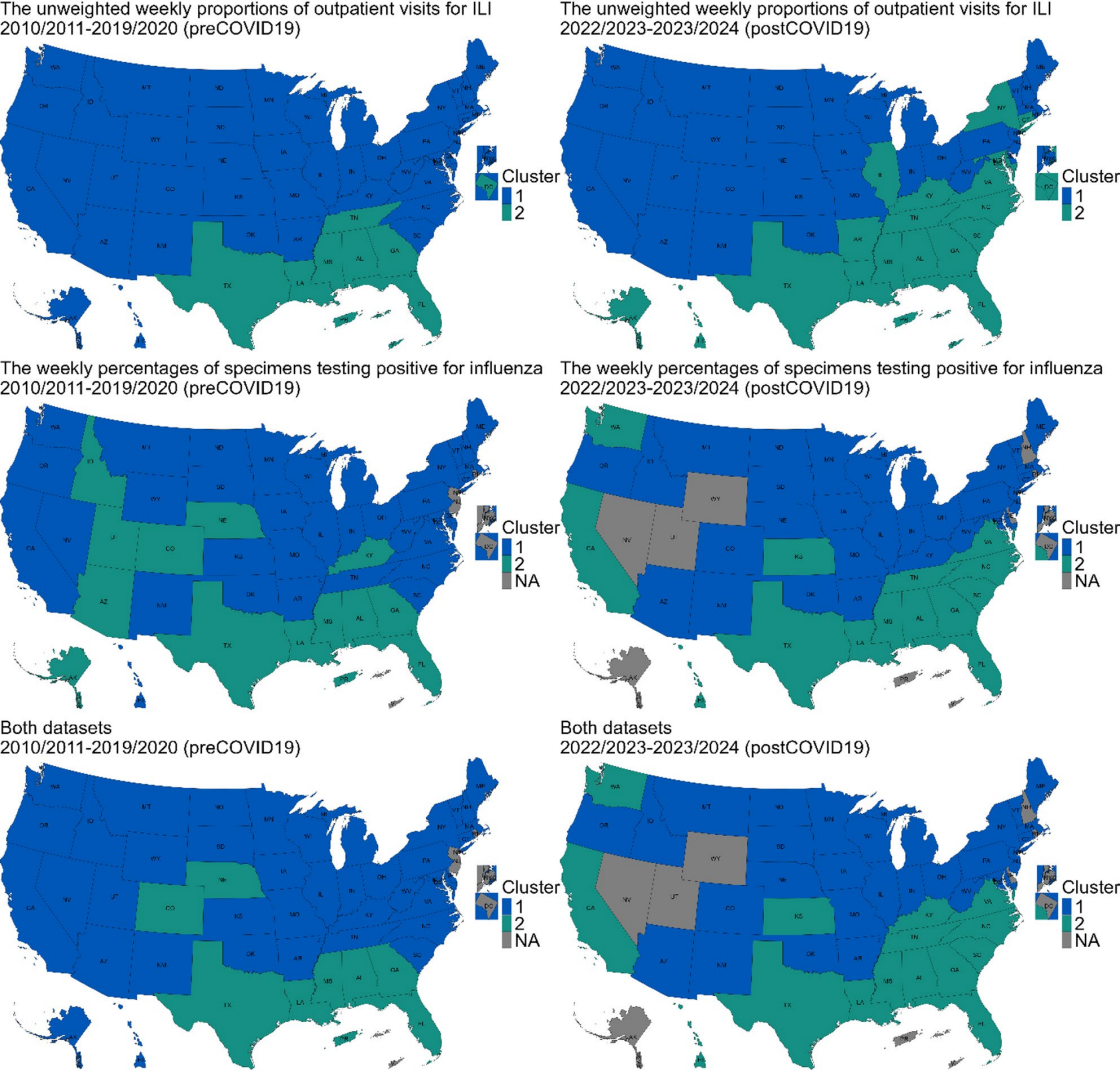
Spatial distribution of clustering patterns for the pre-COVID-19 seasons (2010/2011–2019/2020, left panels) and the post-COVID-19 seasons (2022/2023–2023/2024, right panels). (Upper panels) Univariate analysis based on the unweighted weekly proportions of outpatient visits for ILI, (Middle panels) univariate analysis based on the weekly percentages of specimens testing positive for influenza, and (Lower panels) multivariate analysis using both datasets. The blue color represents the largest cluster containing most jurisdictions, while the green color represents the smaller cluster with fewer jurisdictions. The gray jurisdictions were excluded from the analysis due to insufficient data across all seasons

The time series clustering analysis identified two distinct spatial clusters, indicating that influenza activity across the United States primarily followed two comparable temporal trends (Fig. 2 and Figure S12). Although earlier analyses showed spatial correlation in peak timing, the clustering analysis further showed that some jurisdictions shared similar influenza activity patterns across the entire season. One of the clusters was a Southeastern cluster, which consistently included 5 core members: Georgia (GA), Alabama (AL), Mississippi (MS), Louisiana (LA), and Florida (FL). During the pre-COVID-19 period (2010/2011–2019/2020), the Southeastern cluster also included Texas (TX) and Puerto Rico (PR).

The two univariate analyses, based on different datasets (the unweighted weekly proportions of outpatient visits for ILI and the weekly percentages of specimens testing positive for influenza) presented slightly different patterns. The former included Tennessee (TN) in the Southeastern cluster, as well as the U.S. Virgin Islands (VI) and District of Columbia (DC), while the latter included some Central states such as Colorado (CO) and Nebraska (NE). In the multivariate analysis using both datasets, Colorado (CO) and Nebraska (NE) remained within the Southeastern cluster. We then verified cluster membership by identifying jurisdictions within one standard deviation from their respective centroids and found only a limited number of outliers (Figure S13). Notably, the clustering patterns of the 5 core Southeastern members remained robust during the post-COVID-19 seasons (2022/2023–2023/2024) (Fig. 2).

**Fig. 3:**
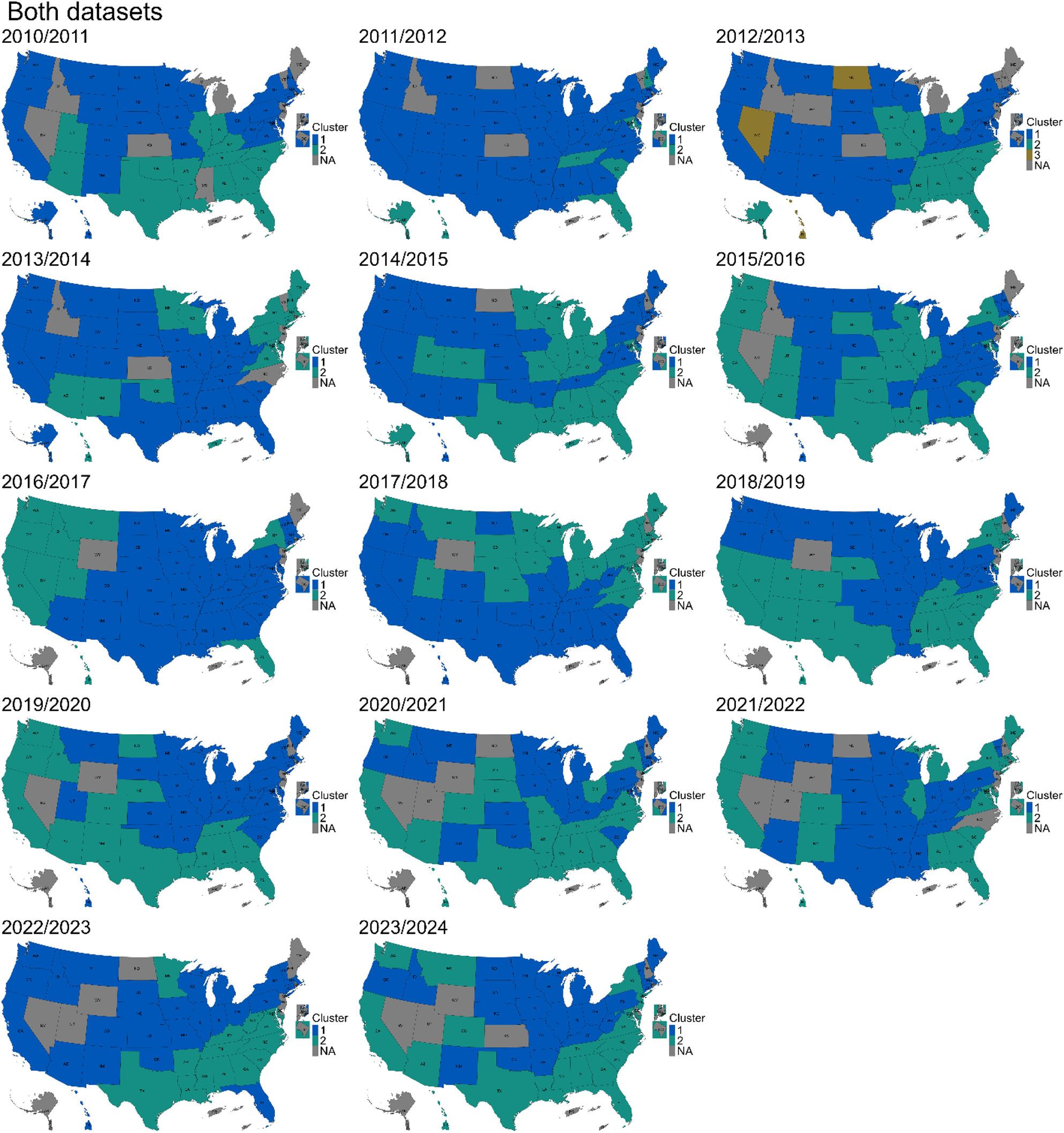
Spatial distribution of clustering patterns from the 2010/2011 to 2023/2024 seasons, based on multivariate analysis using both datasets (the unweighted weekly proportions of outpatient visits for ILI and the weekly percentages of specimens testing positive for influenza). The latter consist of both public health and clinical laboratory data reported before the 2015/2016 season, while only the clinical laboratory data were available from the 2015/2016 season onward. The blue color represents the largest cluster containing most jurisdictions, while the green color represents the smaller cluster with fewer jurisdictions. In the 2012/2013 season, three clusters were identified, represented by blue, green, and brown colors, corresponding to the largest, second smallest, and smallest clusters, respectively. The gray jurisdictions were excluded from the analysis due to insufficient data for the respective seasons

Clustering patterns demonstrated inter-season variability throughout the study period, which was assessed by comparing cluster membership across individual seasons (Fig. 3, Figure S14 and Figure S15). While the core Southeastern members generally maintained consistent cluster associations, some other jurisdictions experienced significant season-to-season changes in cluster membership, highlighting the dynamic nature of influenza activity. The Western and Central states showed the greatest variability, with shifts between clusters from one season to the next.

### Significant differences across clusters in local Moran’s I and proportion of A/H1 viruses

During the pre-COVID-19 seasons (2010/2011–2019/2020), the ANOVA revealed significant differences across clusters in local Moran’s I (*p* < 0.001) and the proportion of all influenza A virus detections that were influenza A/H1 viruses (*p* < 0.001). The Tukey HSD test confirmed that the smaller cluster with fewer jurisdictions exhibited significantly higher spatial autocorrelation (mean difference in local Moran’s I = 0.16) compared to the larger cluster. This suggests that influenza activity within the smaller cluster (typically the Southeastern cluster) was more geographically synchronized. Similarly, the Tukey HSD test indicated that the smaller cluster had a significantly higher proportion of all influenza A virus detections that were influenza A/H1 viruses (mean difference = 2%) compared to the larger cluster. Although the magnitude of the difference was relatively small, this indicates potential spatial heterogeneity in viral circulation across regions.

In contrast, we found no significant differences across clusters in peak timing (*p* = 0.825) or the proportion of all influenza A and B virus detections that were influenza A viruses (*p* = 0.0623). However, significant interactions between clusters and seasons indicated that temporal variability may also influence these relationships (Table S2). Notably, seasonal variation emerged as a dominant factor influencing all four dependent variables: peak timing, local Moran’s I, the proportion of all influenza A and B virus detections that were influenza A viruses, and the proportion of all influenza A virus detections that were influenza A/H1 viruses (all *p* < 0.001). This suggests that seasonal variability in influenza activity played a key role in shaping spatial patterns and aligns with the inter-season variability identified in earlier sections. Further details are provided in the supplementary materials (Figure S17, Figure S18, Figure S19, Figure S20, and Table S2).

## Discussion

### Consistent spatial clustering patterns of seasonal influenza

Our study reveals distinct spatial clustering patterns of seasonal influenza in the United States. We found a consistent grouping of Southeastern states, particularly Georgia, Alabama, Mississippi, Louisiana, and Florida, suggesting a specific spatiotemporal pattern of seasonal influenza for this region that was also reflected in earlier seasonal peaks and high local spatial autocorrelation. Notably, earlier peak timing indicates that influenza activity reaches its maximum earlier in the season, but does not necessarily imply earlier epidemic onset or longer epidemic duration. However, prior studies have also documented earlier seasonal influenza activity in the Southern United States [11, 12].

Our findings highlight regional variations in influenza dynamics that could have implications for public health strategies. The consistent clustering observed in Southeastern states suggests that these areas tend to experience earlier influenza activity on average. Recognizing these patterns can help inform influenza prevention and control efforts, including timing of vaccination campaigns and public communication. At the same time, knowledge of established spatial clustering can support regional situational awareness by allowing surveillance efforts to interpret local influenza trends in the context of activity observed in other jurisdictions within the same cluster. For example, increases in influenza activity in one Southeastern jurisdiction may provide early contextual information about expected trends in other clustered jurisdictions, which may be particularly valuable when surveillance data are sparse, delayed, or temporarily unavailable.

More broadly, recognizing spatial clustering can inform expectations about where similar transmission dynamics are likely to occur and guide the interpretation of surveillance signals by explicitly incorporating spatial context rather than treating jurisdictions as independent units. However, occasional shifts in cluster membership among Western and Central states, as well as the presence of outliers, emphasizes the continued importance of considering local factors that influence influenza activity. Maintaining robust surveillance and long-term monitoring across regions should provide valuable insights for refining broader public health responses to seasonal influenza.

### Seasonal variations

The variation in clustering patterns observed across individual seasons underscores the dynamic nature of influenza activity. These season-to-season variations, as seen in the significant interactions between clusters and seasons, highlight the inherent challenges in forecasting influenza trends. Our findings closely align with the earlier work of Rosensteel et al. [20], who characterized significant heterogeneity in influenza activity across seasons during the 2002–2009 period, and Dahlgren et al. [21], who found substantial variation during the 2010–2016 seasons. Our results demonstrate that this spatiotemporal heterogeneity continued in the post-2009 pandemic era and following the COVID-19 pandemic, e.g., in the 2022/2023 and 2023/2024 seasons.

Longer-term analyses are needed to fully understand the impact of COVID-19 pandemic on influenza activity patterns. These disruptions are likely due to changes in population behavior, non-pharmaceutical interventions, and reduced exposure to influenza viruses during the period. For instance, the impact of school openings on influenza spread has been well-documented, particularly during the 2009 pandemic [18]. Additionally, the emergence of antigenically drifted influenza viruses and changes in genetic clades can further alter epidemic patterns and severity. Integrating such insights with longer time series data could provide a more comprehensive understanding of the shifts observed during and after the COVID-19 pandemic.

### Data variability

Our analysis revealed some variations in cluster membership by data source, particularly for the Western and Central states, which occasionally showed similarities to the Southeastern cluster when using the weekly percentages of specimens testing positive for influenza. The data on specimens testing positive for influenza virus were limited by missing values, particularly during the summer months, which may affect the robustness of our analysis for some jurisdictions. Additionally, outpatient visits for ILI are defined syndromically and lack laboratory confirmation, and therefore capture activity of other respiratory viruses in addition to influenza-specific activity. In contrast, virologic surveillance reflects laboratory-confirmed influenza virus detections. As a result, these indicators provide complementary perspectives on influenza activity, each with distinct strengths and limitations [32, 33]. Within the test positivity data, the public health and clinical laboratory data differ in surveillance coverage and purpose. The former reflects routine diagnostic testing and is better suited for assessing the timing and intensity of influenza circulation, whereas the latter is based on targeted sampling for virologic characterization. The analyses were therefore structured to reflect these differences in surveillance design and data availability across systems and seasons.

Despite these challenges, our univariate analyses provide unique insights from each data source. For example, using the unweighted weekly proportions of outpatient visits for ILI allowed us to explore patterns for the U.S. Virgin Islands and the District of Columbia, even though these were not included in the weekly percentages of specimens testing positive for influenza virus. We also performed a multivariate approach to combine information from both datasets and confirm the identification of the Southeastern cluster. However, as these data are based on healthcare visits and testing, they are subject to potential biases related to healthcare access and care-seeking behaviors [33].

In this study, our primary objective was to characterize spatial clustering patterns and to provide an initial quantitative comparison of selected characteristics across clusters, rather than to identify specific causal drivers. To this end, we used ANOVA to assess differences in the proportion of influenza A/H1 viruses across clusters, illustrating how cluster-based analyses can be used to examine potential epidemiologic effects associated with regional patterns.

Geographic proximity itself is not a direct causal driver of influenza activity, but rather serves as a proxy for underlying factors that vary spatially and influence transmission dynamics. Jurisdictions that are geographically proximate are more likely to share similar meteorological conditions, population demographics, contact and mobility patterns, vaccination coverage, and levels of prior immunity, which can contribute to correlated influenza activity and similar epidemic timing. Additional data sources including climate variables, environmental factors, population density, and socioeconomic indicators may help explain observed deviations from spatial clustering. For example, earlier studies [15, 16] demonstrated that humidity plays a key role in seasonal influenza transmission and Dalziel et al. [14] further highlighted that humidity is a significant driver particularly in urban areas. Extending our work to incorporate such variables could offer a broader understanding of their influence on influenza spatial transmission dynamics.

### State and territory level heterogeneity

Our analysis was limited to state and territory levels, which may mask finer-scale heterogeneity in influenza activity patterns. County-level analysis, such as in Rosensteel et al. [20], and studies of core-based statistical areas, such as Dahlgren et al. [21], could reveal additional important patterns, especially in densely populated urban areas. One key factor contributing to such finer-scale heterogeneity is human mobility. While state and territory level analyses offer broad regional insights, influenza transmission is heavily influenced by movement patterns within and between cities, commuting zones, and rural areas [12, 17]. Future studies integrating mobility data, such as commuting flows and other travel behaviors, and examining their effects in both urban and rural settings, could enhance our understanding of spatial transmission patterns.

## Conclusions

This study quantified distinct spatial differences in seasonal influenza activity across the United States. Our results suggest that interpreting national influenza trends may overlook important regional variations. For example, the Southeastern United States, including Georgia, Alabama, Mississippi, Louisiana, and Florida, often experienced earlier influenza activity compared to other regions, although these spatiotemporal patterns were not consistent across seasons, reflecting the dynamic nature of influenza transmission. More generally, our findings emphasize the importance of robust state-based influenza surveillance systems, like those supported by CDC, to provide actionable information for understanding regional influenza dynamics. Adaptive approaches and stable long-term surveillance data are critical for effectively addressing regional differences in influenza activity. The potential impacts of variation in seasonal timing should be considered when planning influenza prevention and control efforts, including timing of vaccination campaigns and public communication, to enhance preparedness and effectiveness nationwide. 

## Electronic Supplementary Material

Below is the link to the electronic supplementary material.

Supplementary Material 1

## Data Availability

The surveillance data utilized in this study are publicly available through the CDC’s FluView Interactive platform (https://gis.cdc.gov/grasp/fluview/fluportaldashboard.html). All analyses were performed using R version 4.4.0. The R scripts and code supporting the findings of this study are accessible on GitHub at https://github.com/CDCgov/influenza-cluster-us.

## Declarations

### Declaration of using large language models in the writing process

During the preparation of this work the authors used CDC AI Chatbot (GPT-4o) in order to enhance the clarity, coherence, and correctness of the writing, and to check for grammatical errors. After using this tool, the authors reviewed and edited the content as needed and take full responsibility for the content of the publication.
