# Supplementary material for: Understanding spatiotemporal clustering of seasonal influenza in the United States
Source: BMC Infect Dis. 2026 Mar 4;26:746. doi: 10.1186/s12879-026-13000-7 (PMC13067465; doi:10.1186/s12879-026-13000-7)
Supplement: Supplementary file 1 — Supplementary Material 1 [file 12879_2026_13000_MOESM1_ESM.docx]

Understanding spatiotemporal clustering of seasonal influenza in the United States

Louis Yat Hin Chan^1,*^, Sinead Morris^1,2^, Norman Hassell^1^, Perrine Marcenac^1^, Alexia Couture^1^, Arielle Colon^1^, Krista Kniss^1^, Alicia Budd^1^, Matthew Biggerstaff^1^, Rebecca Borchering^1^

1. Influenza Division, Centers for Disease Control and Prevention, Atlanta, Georgia, USA
2. Goldbelt Professional Services, Chesapeake, Virginia, USA

* Influenza Division, Centers for Disease Control and Prevention, 1600 Clifton Road, Atlanta, GA 30329, USA ([LouisChan@cdc.gov](mailto:LouisChan@cdc.gov))

# Supplementary material

## Surveillance data

For the 2020/2021–2023/2024 seasons, data availability differed from the pre-COVID-19 period (Figure S1, Figure S2 and Figure S3). The unweighted weekly proportions of outpatient visits for ILI were available for all jurisdictions. However, the weekly percentages of specimens testing positive for influenza included fewer jurisdictions compared to the pre-COVID-19 seasons, though the jurisdictions included in the analysis exhibited fewer missing data (Table S1). Importantly, influenza activity levels during the 2020/2021 season were notably lower, reflecting the widespread impact of the COVID-19 pandemic.

Figure S1 and Figure S2 show the weekly surveillance data before and after imputation and smoothing, while Figure S3 shows the corresponding processed data, used in the main analyses. Together, these figures provide transparency regarding the data processing steps and their impact on the time series.

Missing weekly values were imputed using Stineman interpolation, as described in the Methods. To reduce short-term noise and better capture seasonal patterns, the imputed weekly time series were smoothed using Nadaraya–Watson kernel regression.

For each week $w$, the smoothed value $\hat{y}(w)$ was estimated as a locally weighted average of observed values in neighboring weeks, given by $\hat{y}(w)=\frac{\sum_{w'} K(\frac{w-w^{'}}{h})y(w')}{\sum_{w'} K(\frac{w-w'}{h})}$, where $y(w')$ denotes the observed value at week $w'$, $K(\cdot)$ is the kernel function, and $h$ is the bandwidth parameter controlling the temporal smoothing scale.

We used a Gaussian kernel, $K(u)=e^{-u^{2}/2}$, which assigns greater weight to observations closer in time. As in standard Nadaraya–Watson estimation, the normalization constant of the Gaussian kernel cancels in the weighted average and is therefore omitted.

We set the bandwidth to $h=4$ weeks, corresponding to a smoothing scale of approximately one month. This choice reflects a balance between reducing high-frequency variability in weekly surveillance data and preserving the overall seasonal structure of influenza epidemics.

| **Study period** | **The unweighted weekly proportions of outpatient visits for ILI** | | **The weekly percentages of specimens testing positive for influenza** | |
| --- | --- | --- | --- | --- |
|  | Number of jurisdictions | Proportion of available data | Number of jurisdictions | Proportion of available data |
| **pre-COVID-19 season** | | | | |
| 2010/2011 | 52 | 1.00 | 41 | 0.87 |
| 2011/2012 | 53 | 1.00 | 44 | 0.87 |
| 2012/2013 | 53 | 1.00 | 42 | 0.89 |
| 2013/2014 | 54 | 1.00 | 45 | 0.90 |
| 2014/2015 | 54 | 1.00 | 47 | 0.94 |
| 2015/2016 | 54 | 1.00 | 44 | 0.96 |
| 2016/2017 | 54 | 1.00 | 45 | 0.95 |
| 2017/2018 | 54 | 1.00 | 45 | 0.96 |
| 2018/2019 | 54 | 1.00 | 45 | 0.97 |
| 2019/2020 | 54 | 1.00 | 44 | 0.96 |
| **COVID-19-era season** | | | | |
| 2020/2021 | 54 | 1.00 | 41 | 0.98 |
| 2021/2022 | 54 | 1.00 | 40 | 0.98 |
| **post-COVID-19 season** | | | | |
| 2022/2023 | 54 | 1.00 | 40 | 0.99 |
| 2023/2024 | 54 | 1.00 | 42 | 0.99 |

Table S1. Number of jurisdictions included in the analysis after data processing, along with the proportion of available data for the jurisdictions included.


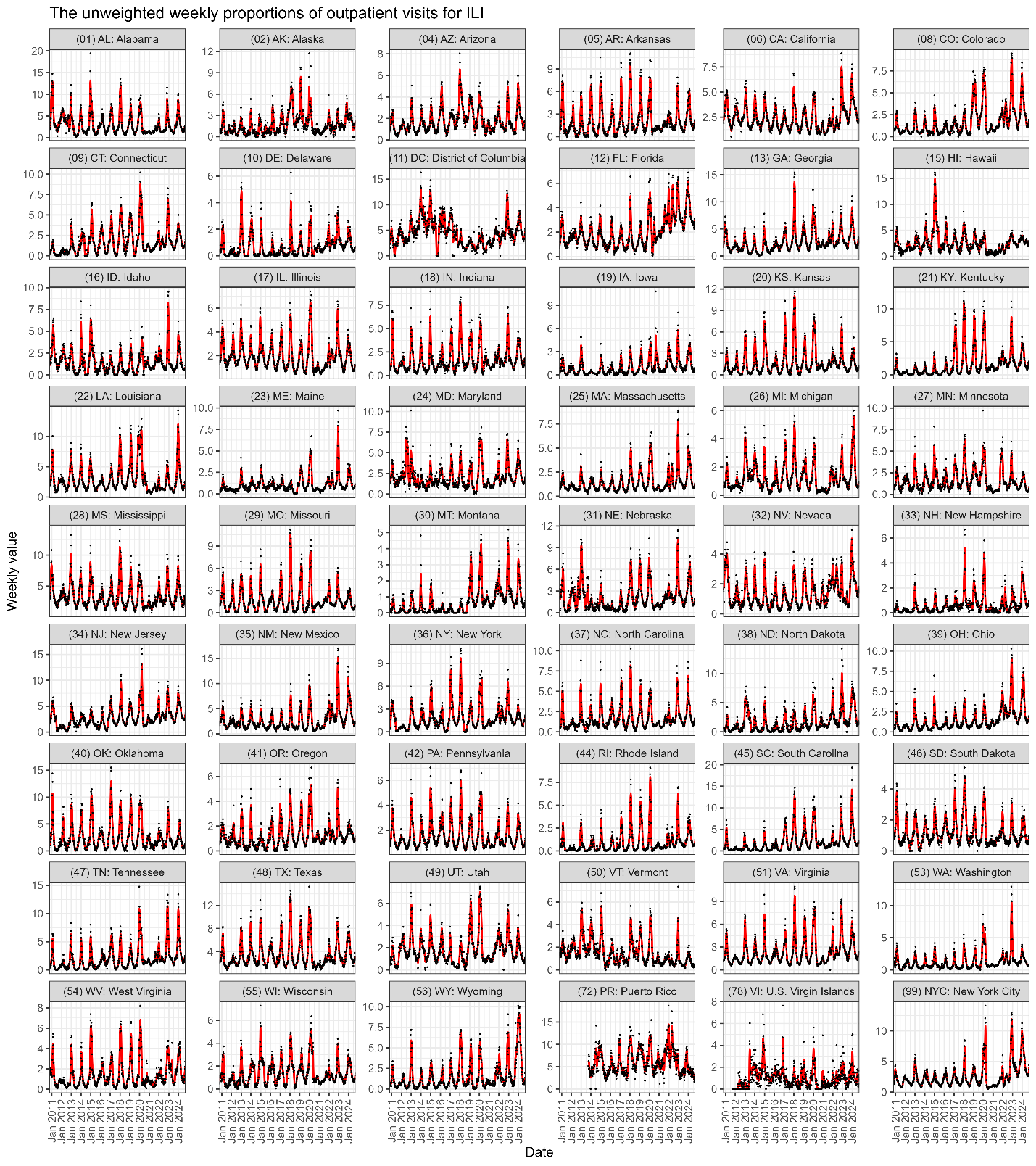


Figure S1. The unweighted weekly proportions of outpatient visits for ILI. Black points represent the observed weekly values, and red lines indicate the imputed and smoothed series used in the main analysis, obtained using a kernel smoothing bandwidth of 4 weeks. White space represents jurisdictions with more than 50% missing data, which were excluded from the analysis due to insufficient data for the respective seasons.


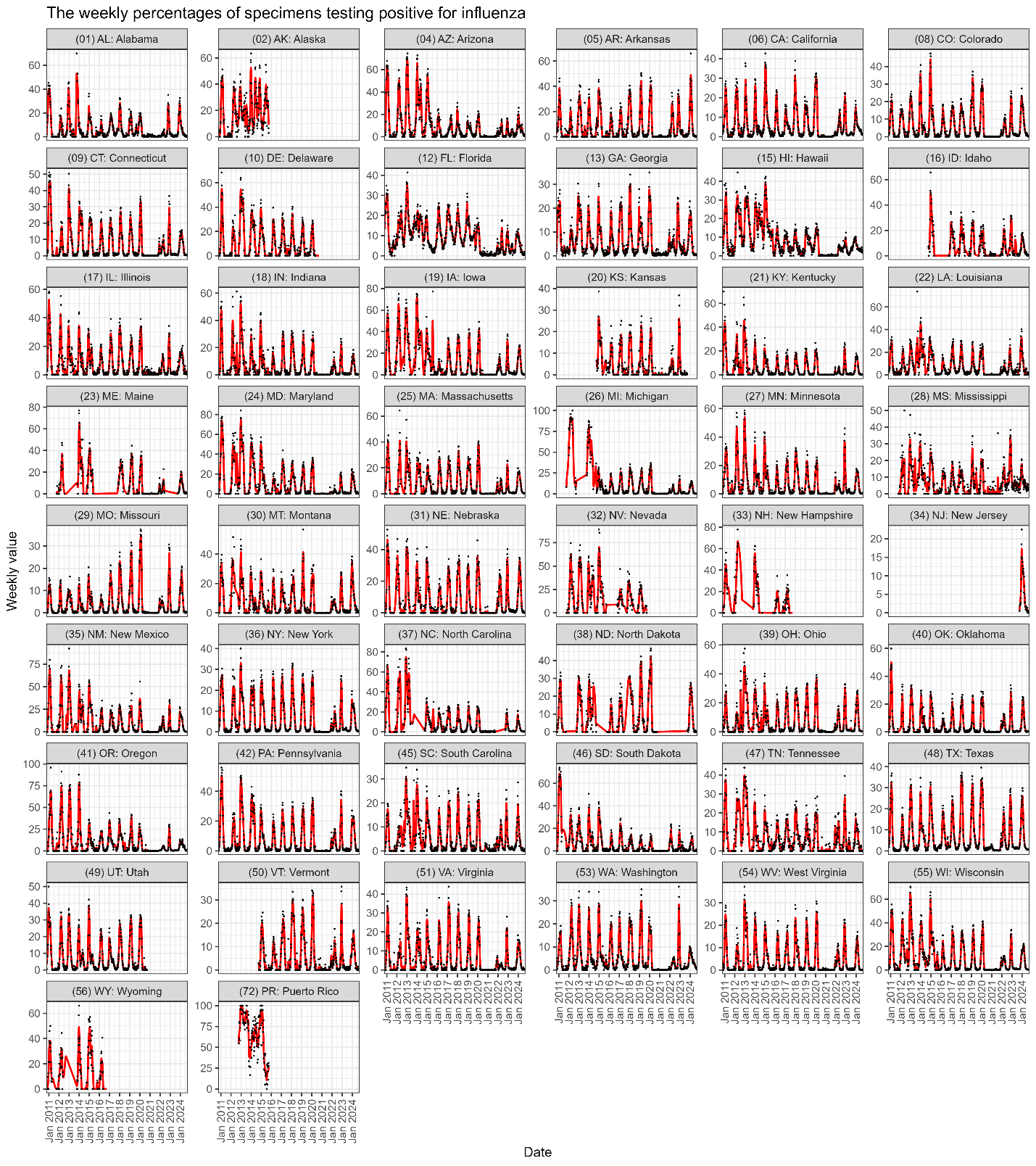


Figure S2. The weekly percentages of specimens testing positive for influenza. The data consist of both public health and clinical laboratory data reported before the 2015/2016 season, while only the clinical laboratory data were available from the 2015/2016 season onward. Black points represent the observed weekly values, and red lines indicate the imputed and smoothed series used in the main analysis, obtained using a kernel smoothing bandwidth of 4 weeks. White space represents jurisdictions with more than 50% missing data, which were excluded from the analysis due to insufficient data for the respective seasons.


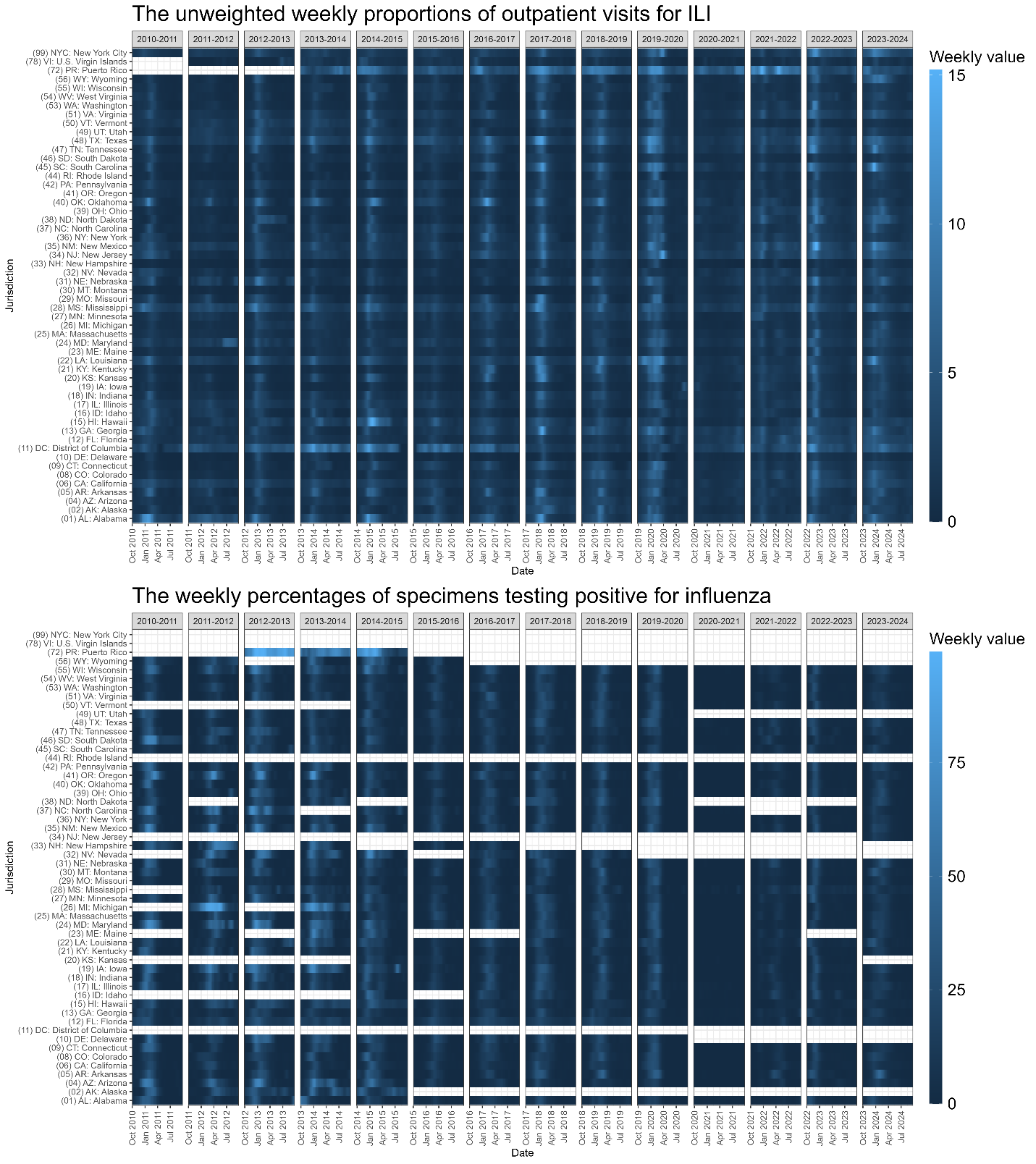


Figure S3. Weekly values of the unweighted weekly proportions of outpatient visits for ILI and the weekly percentages of specimens testing positive for influenza. The latter consist of both public health and clinical laboratory data reported before the 2015/2016 season, while only the clinical laboratory data were available from the 2015/2016 season onward. All weekly values were processed by imputation and smoothing. The color scale ranges from dark to light blue, indicating values from low to high. White space represents jurisdictions with more than 50% missing data, which were excluded from the analysis due to insufficient data for the respective seasons.

## Comparison of peak timing from averaged and single-season time series

Peak timing can be defined in multiple ways. Estimates derived from an averaged time series are not necessarily equivalent to summaries of peak timing obtained from individual seasons. In the main analysis, we defined peak timing using the peak week of the averaged time series. This choice was made for consistency with the clustering analysis, which used the averaged time series as an input. However, this approach differs conceptually from estimating peak timing separately for each season and summarizing those estimates across seasons.

To characterize these differences, we compared peak timing derived from two approaches for each jurisdiction. In the first approach, weekly values were averaged across seasons (pre-COVID-19 or post-COVID-19) to construct an averaged time series, and the peak week was identified from this averaged profile. This method emphasizes the dominant seasonal structure and reduces the influence of inter-seasonal variability. In the second approach, peak weeks were identified independently for each individual season, and the median of these season-specific peak weeks was calculated for each jurisdiction. This approach preserves inter-seasonal variability in peak timing and reflects the central tendency of observed seasonal peaks. The interquartile range (IQR) of single-season peak weeks was also calculated to summarize variability across seasons.

Comparisons were conducted separately for the unweighted weekly proportions of outpatient visits for ILI and the weekly percentages of specimens testing positive for influenza, and for pre-COVID-19 and post-COVID-19 seasons.

Overall, peak timing estimates from the two approaches were similar. However, in the post-COVID-19 seasons, the averaged time series tended to peak earlier than the median of single-season peaks in several jurisdictions. When weekly values were averaged across such seasons with different peak timing, later peaks were smoothed, resulting in an earlier peak in the averaged time series. These differences are illustrated in Figure S4, Figure S5, and Figure S6.


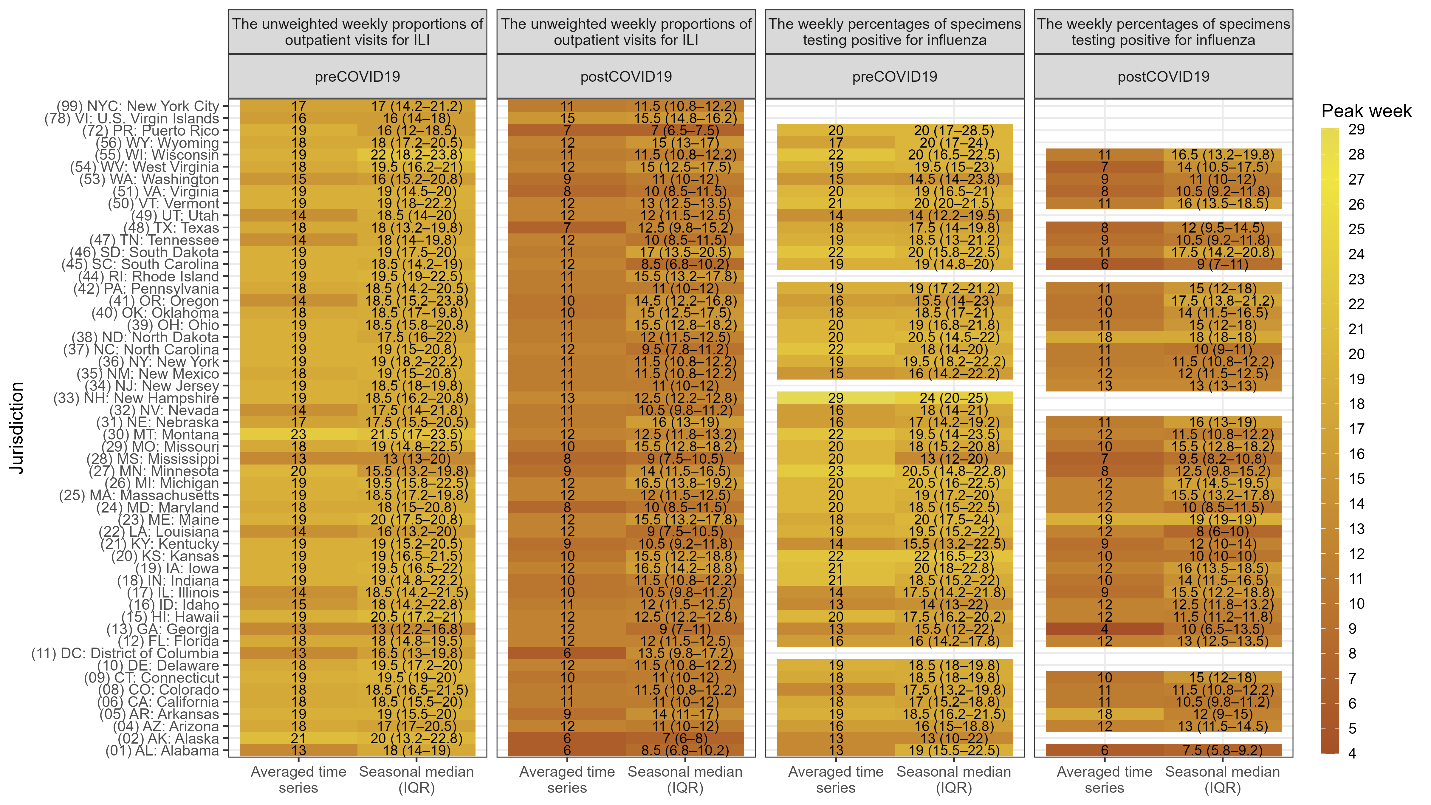


Figure S4. Comparison of peak timing from averaged and seasonal time series. Heatmaps summarizing peak week estimates obtained using two approaches: (i) the peak week identified from the averaged time series and (ii) the median peak week across individual seasons, with the interquartile range (IQR) shown in parentheses. Results are shown separately for pre-COVID-19 and post-COVID-19 seasons and for the unweighted weekly proportions of outpatient visits for ILI and the weekly percentages of specimens testing positive for influenza. Tile colors correspond to the peak week estimate, with averaged time-series peaks shown in the “Averaged time series” columns and medians of single-season peaks shown in the “Seasonal median (IQR)” columns.


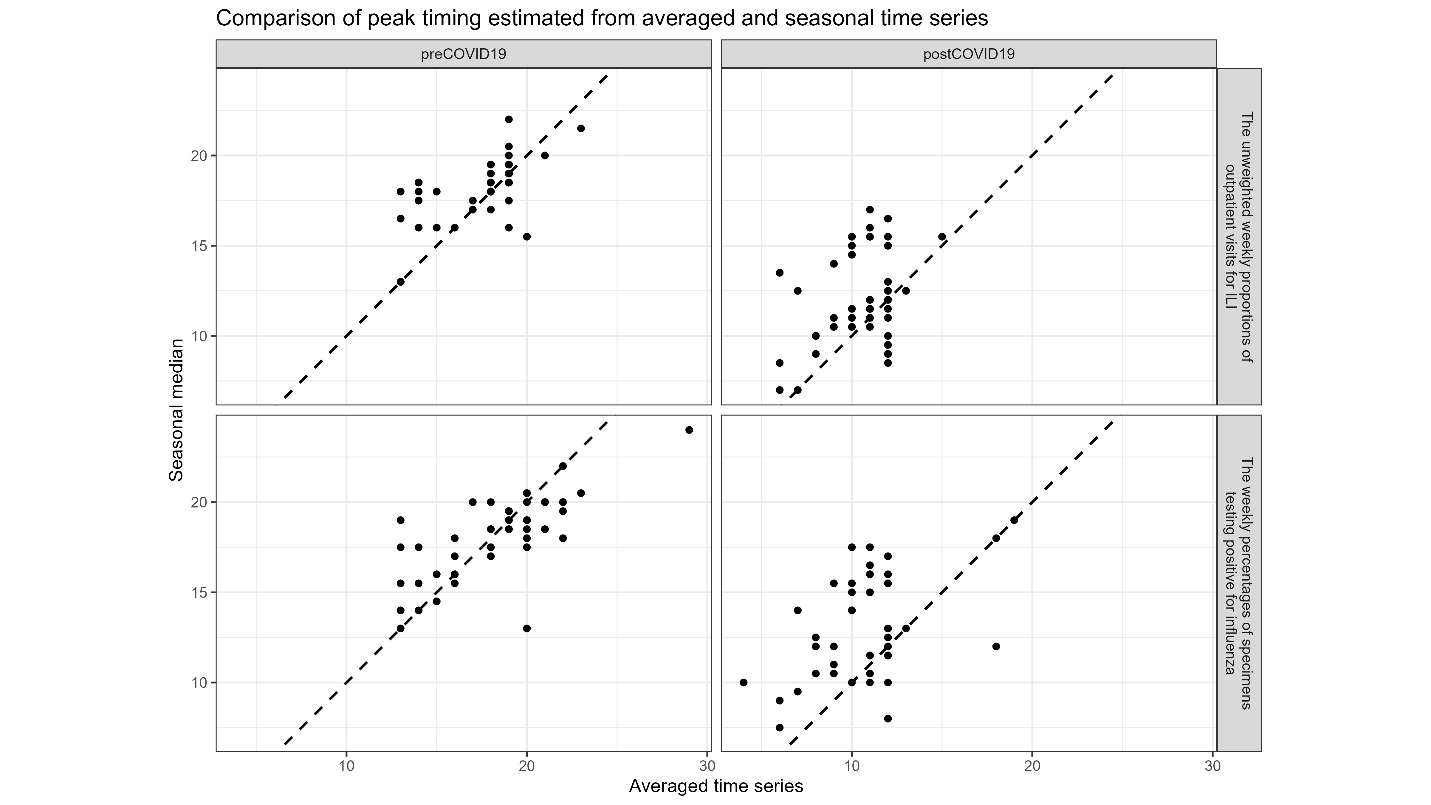


Figure S5. Scatter plots of peak timing from averaged and seasonal time series. Results are shown for pre-COVID-19 (left panels) and post-COVID-19 (right panels) seasons and for the unweighted weekly proportions of outpatient visits for ILI (upper panels) and the weekly percentages of specimens testing positive for influenza (lower panels). Points represent each jurisdiction. The dashed diagonal line indicates agreement between the two peak timing approaches.


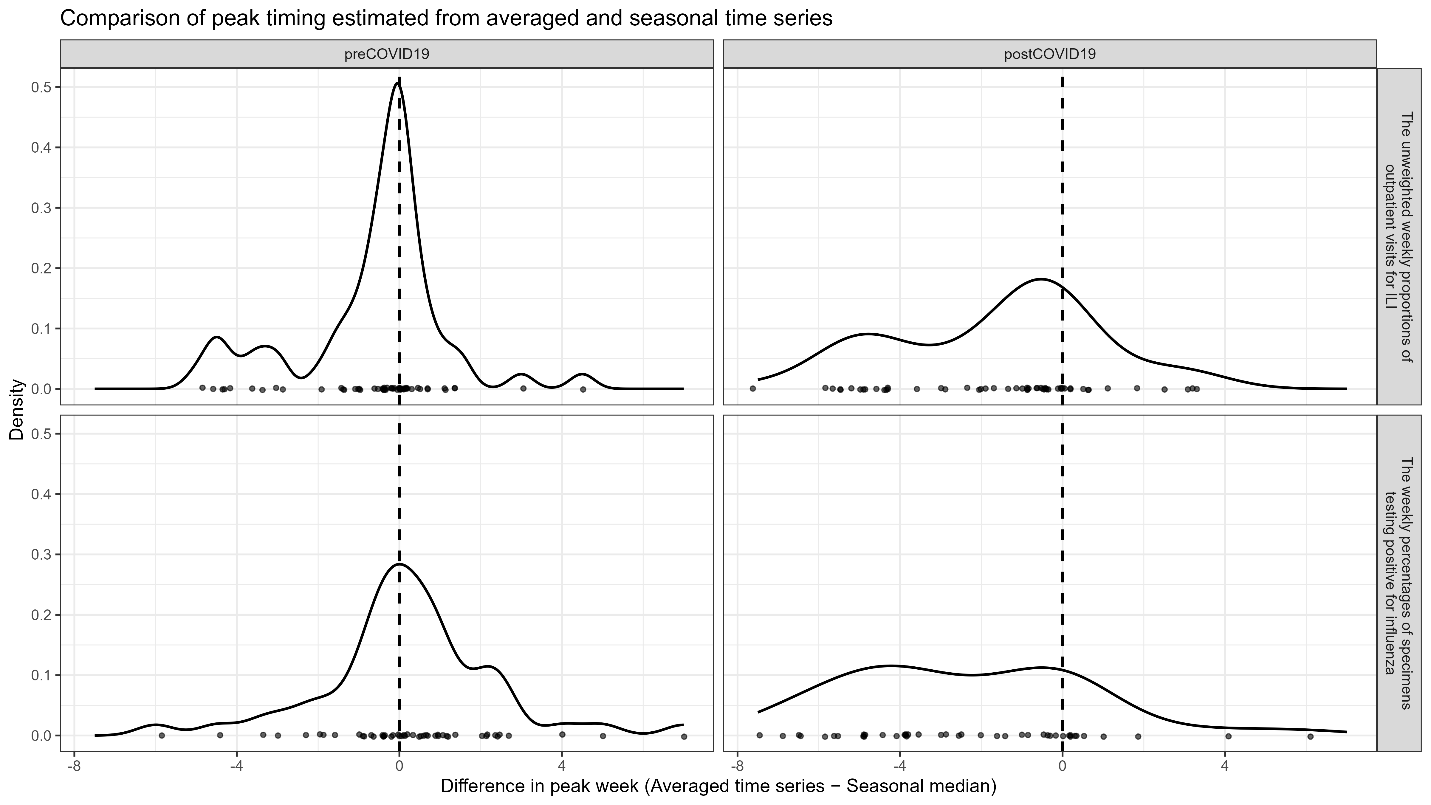


Figure S6. Density plots of peak timing from averaged and seasonal time series. Distributions of differences in peak week, calculated as the peak identified from the averaged seasonal time series minus the median peak week estimated separately from individual seasons. Results are shown for pre-COVID-19 (left panels) and post-COVID-19 (right panels) seasons and for the unweighted weekly proportions of outpatient visits for ILI (upper panels) and the weekly percentages of specimens testing positive for influenza (lower panels). Points along the horizontal axis represent each jurisdiction. The dashed vertical line at zero indicates agreement between the two peak timing approaches.

## Peak timing and Moran’s I


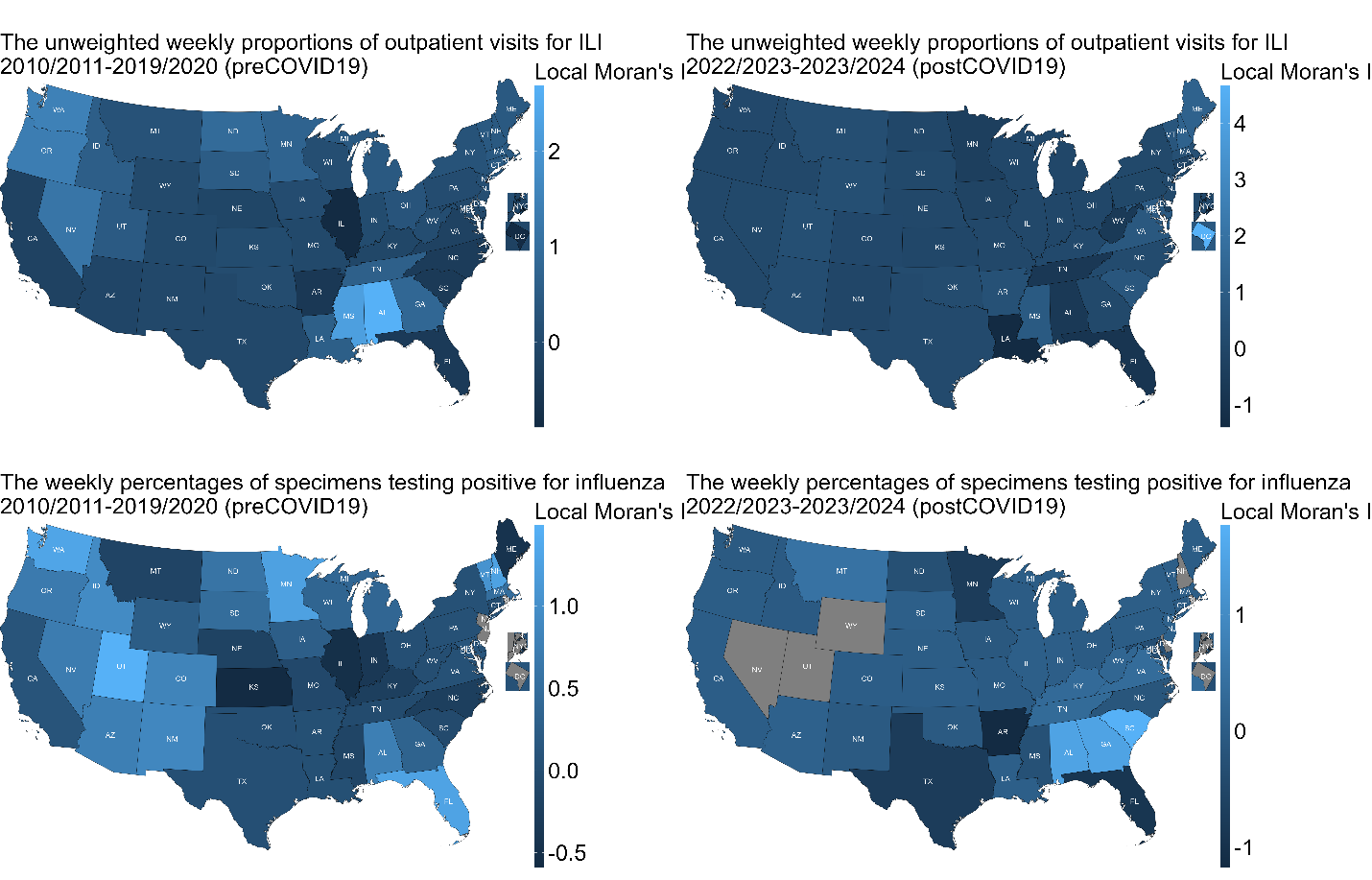


Figure S7. Spatial distribution of local Moran's I values for the unweighted weekly proportions of outpatient visits for ILI (upper panels) and the weekly percentages of specimens testing positive for influenza (lower panels), averaged across the pre-COVID-19 seasons (2010/2011–2019/2020, left panels) and the post-COVID-19 seasons (2022/2023–2023/2024, right panels). The latter consist of both public health and clinical laboratory data reported before the 2015/2016 season, while only the clinical laboratory data were available from the 2015/2016 season onward. Lighter blue indicates higher local Moran's I values (stronger clustering), while darker blue represents lower values. The gray color (e.g., in DC, NJ, and NYC) indicates that all seasons were excluded from the analysis due to missing data.


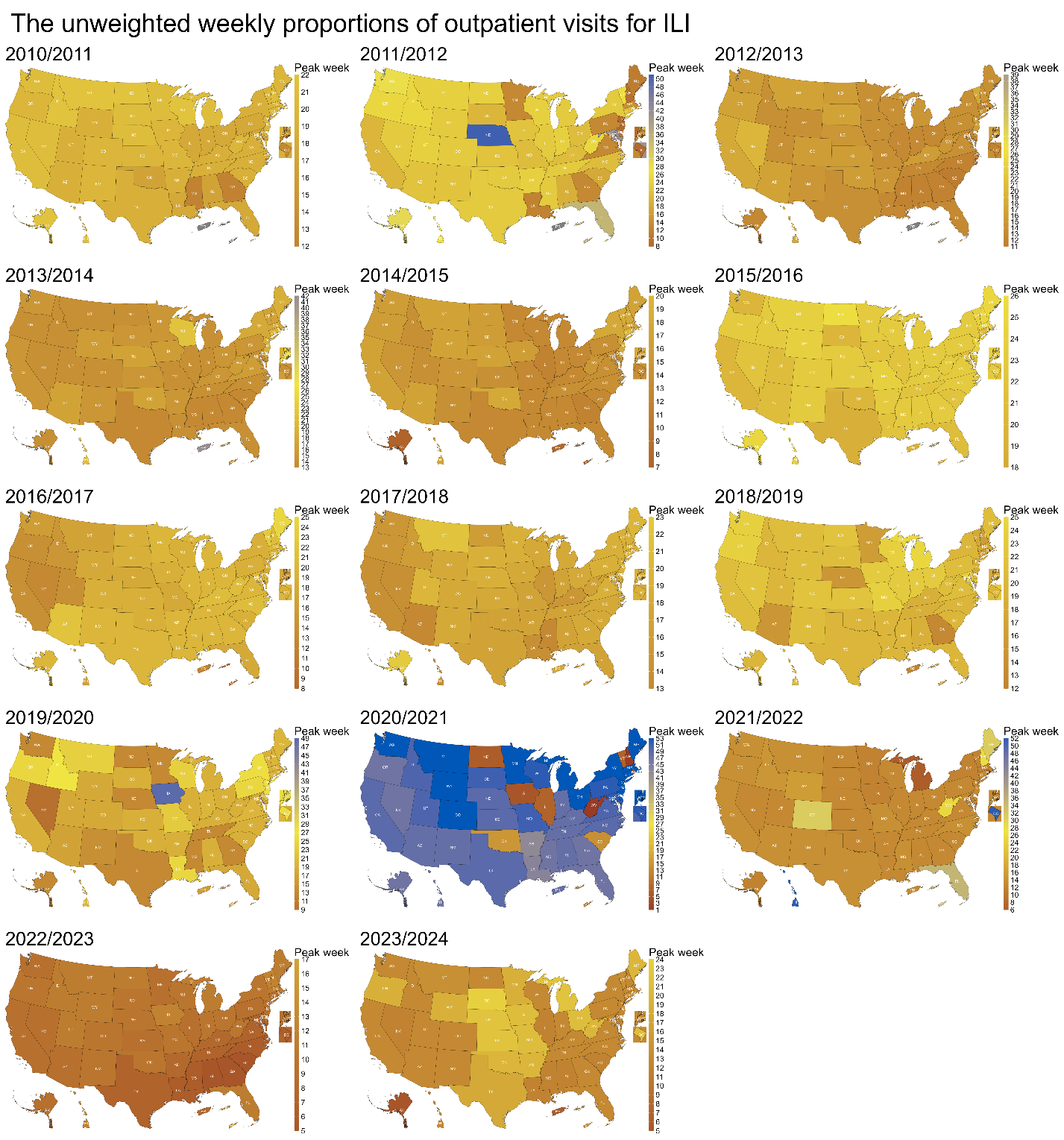


Figure S8. Spatial distribution of peak timing for the unweighted weekly proportions of outpatient visits for ILI, in each season (2010/2011–2023/2024). Darker yellow indicates earlier peak timing, while lighter yellow represents later peak timing. All panels share the same color scale, which corresponds to the week from the beginning of influenza season (MMWR Week 40). The gray color indicates that jurisdictions were excluded from the analysis due to missing data for the respective seasons.


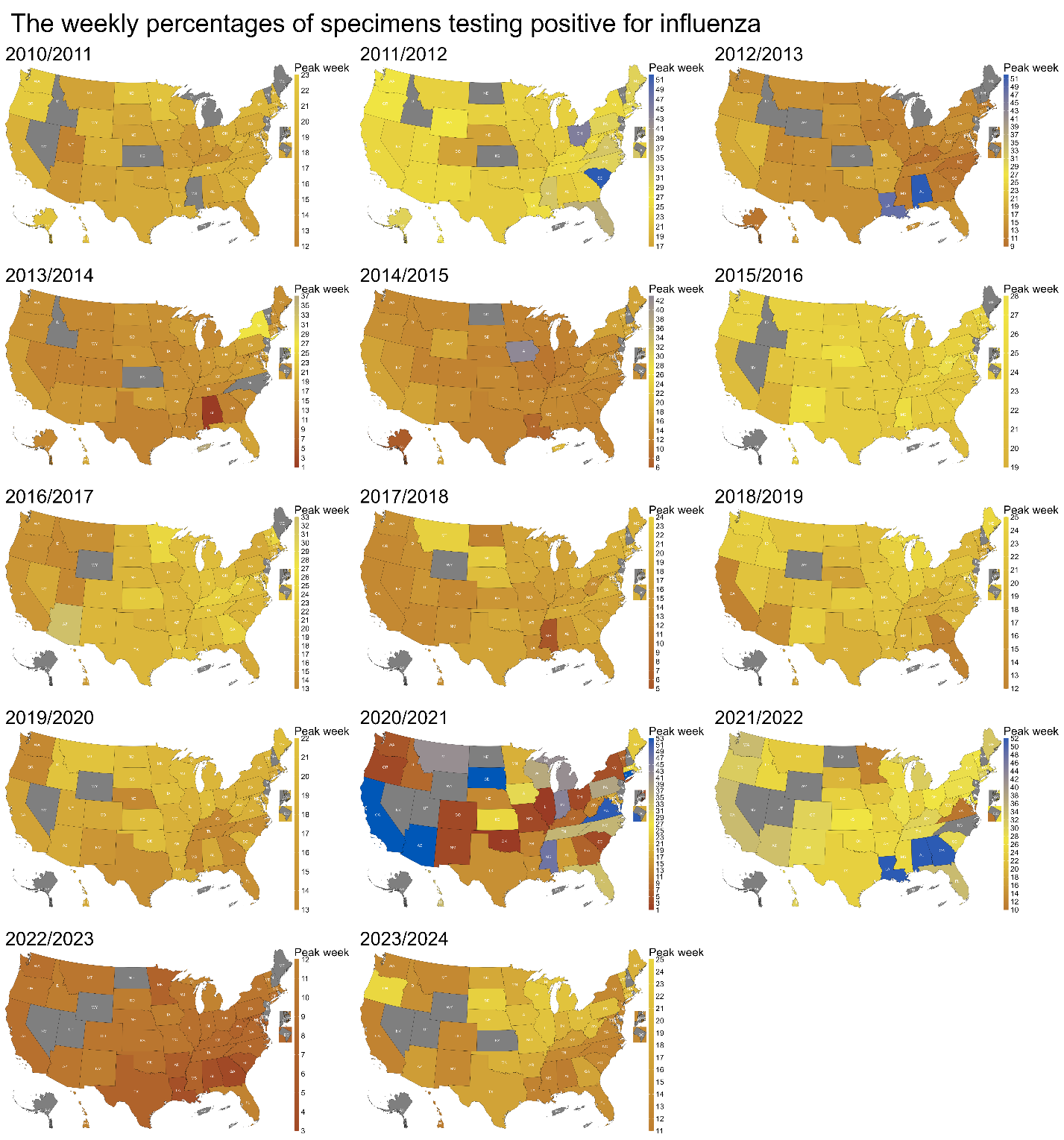


Figure S9. Spatial distribution of peak timing for the weekly percentages of specimens testing positive for influenza, in each season (2010/2011–2023/2024). The data consist of both public health and clinical laboratory data reported before the 2015/2016 season, while only the clinical laboratory data were available from the 2015/2016 season onward. Darker yellow indicates earlier peak timing, while lighter yellow represents later peak timing. All panels share the same color scale, which corresponds to the week from the beginning of influenza season (MMWR Week 40). The gray color indicates that jurisdictions were excluded from the analysis due to missing data for the respective seasons.


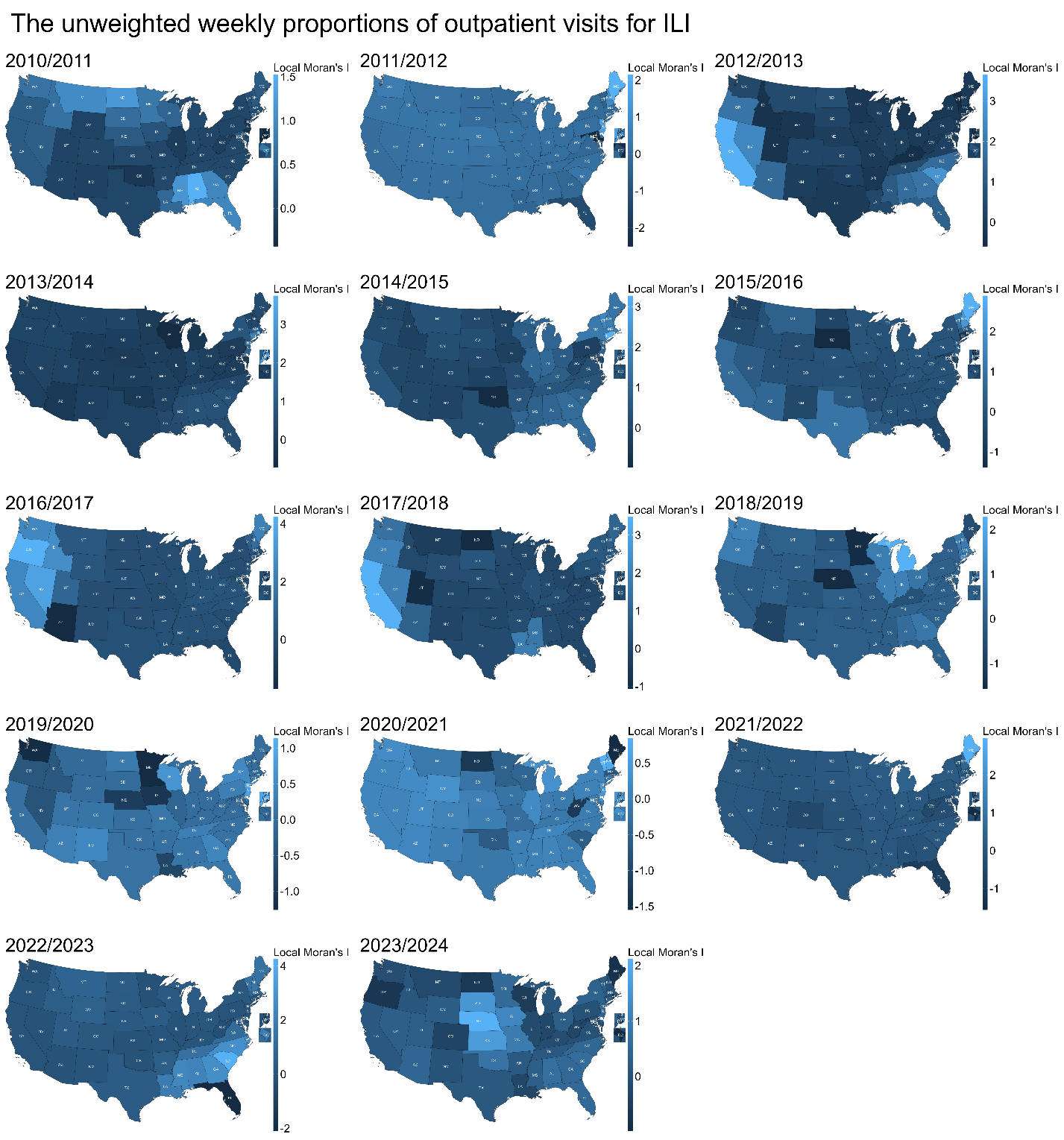


Figure S10. Spatial distribution of local Moran's I values for the unweighted weekly proportions of outpatient visits for ILI, in each season (2010/2011–2023/2024). Lighter blue indicates higher local Moran's I values (stronger clustering), while darker blue represents lower values. The gray color indicates that jurisdictions were excluded from the analysis due to missing data for the respective seasons.


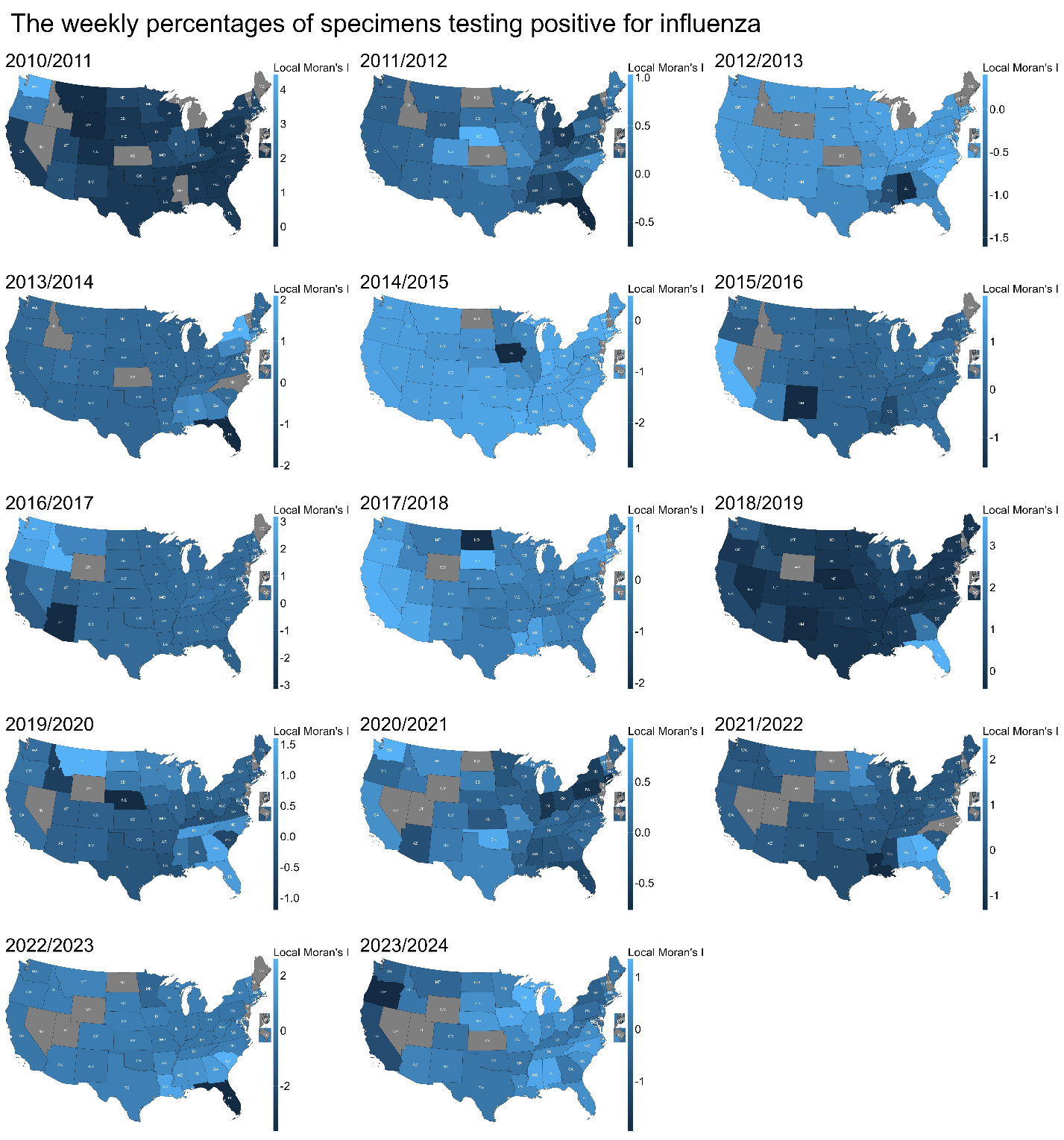


Figure S11. Spatial distribution of local Moran's I values for the weekly percentages of specimens testing positive for influenza, in each season (2010/2011–2023/2024). The data consist of both public health and clinical laboratory data reported before the 2015/2016 season, while only the clinical laboratory data were available from the 2015/2016 season onward. Lighter blue indicates higher local Moran's I values (stronger clustering), while darker blue represents lower values. The gray color indicates that jurisdictions were excluded from the analysis due to missing data for the respective seasons.

## K-means time series clustering analysis

For the time series clustering analysis, we used k-means clustering as implemented in the `dtwclust` R package, with additional details provided here to clarify methodological implementation.

The inputs to the clustering algorithm consisted of jurisdiction-specific time series of influenza activity defined over a given period. Depending on the analysis, clustering was applied either to averaged time series (averaged across seasons within the pre-COVID-19 or post-COVID-19 period) or to individual-season time series. Only jurisdictions with sufficient data for the corresponding datasets were included.

Prior to clustering, each jurisdiction-specific time series within the period was z-normalized independently, so that values within each series were centered to mean zero and scaled to unit variance before comparison with other series. This normalization ensured that clustering was driven by similarities in temporal patterns rather than differences in absolute magnitude.

Clustering was performed using partitional k-means with the Manhattan (L1) distance and mean centroids. The Manhattan distance was chosen to emphasize overall pattern similarity across the season and to reduce sensitivity to localized deviations.

The number of clusters ($k$) was selected using the silhouette method. For a given $k$, the silhouette width for each jurisdiction was calculated based on the L1 distance matrix as $\frac{b_{i}-a_{i}}{\max_{i} (b_{i}-a_{i})}$, where $a_{i}$ is the average distance between jurisdiction $i$ and all other jurisdictions in the same cluster, and $b_{i}$ is the minimum average distance between jurisdiction $i$ and jurisdictions in any other cluster. The silhouette width ranges from −1 to 1, with larger values indicating better separation. We computed silhouette widths for $k$ from 2 to 10 and calculated the silhouette score, defined as the mean silhouette width across all jurisdictions, for each $k$. The value of $k$ that maximized the silhouette score was selected.

Because k-means clustering can be sensitive to initialization, the procedure was repeated 100 times using different random seeds. The clustering solution that was most frequently reproduced across runs was selected as the final result, providing a stable and robust partition of jurisdictions.


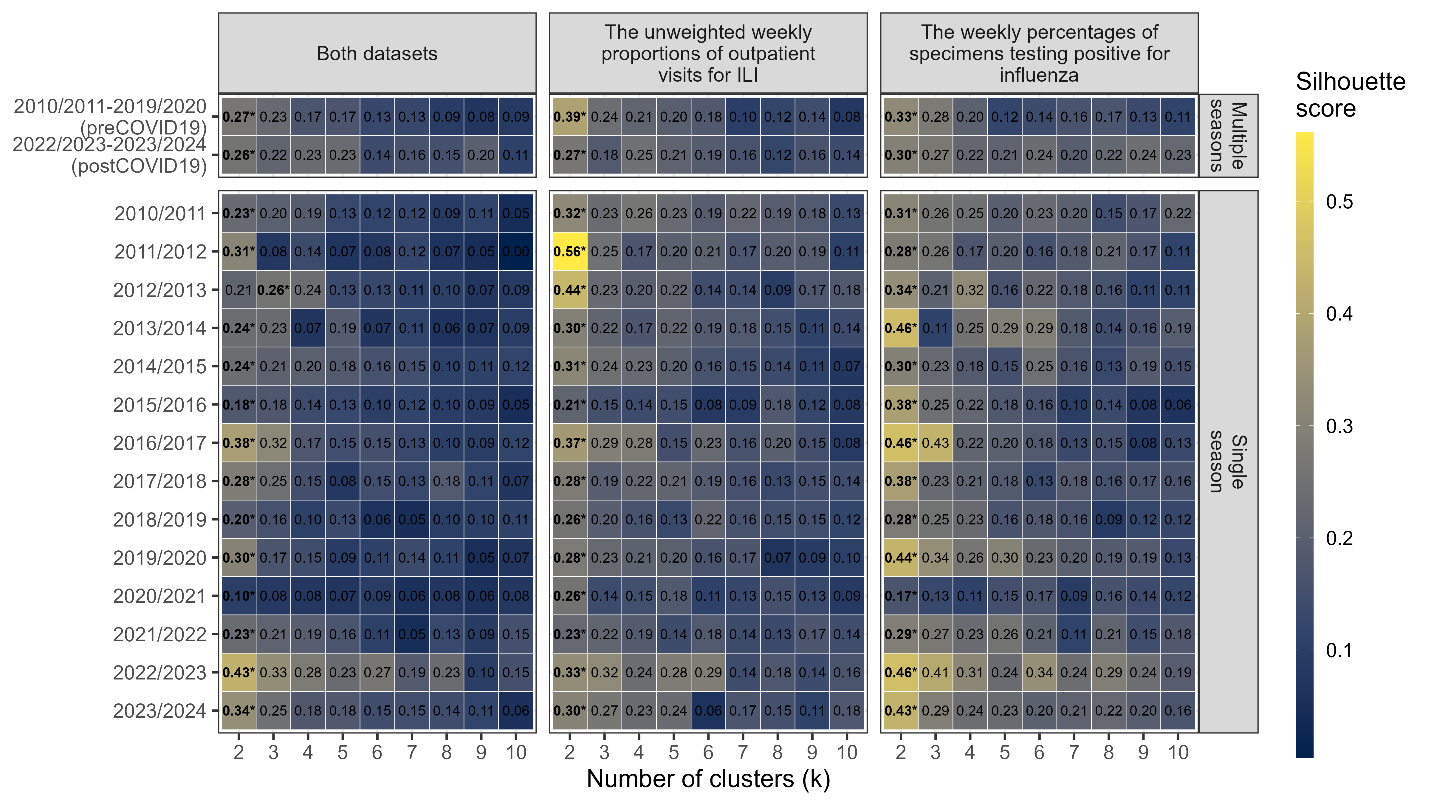


Figure S12. Selection of the number of clusters using the silhouette method. The heatmaps show the silhouette scores for k-means clustering across different numbers of clusters (k) from 2 to 10. The results are shown separately for univariate and multivariate analyses and for multiple-season averaged and single-season time series. For each panel, values represent the mean silhouette scores averaged across all jurisdictions, with higher values indicating better separation between clusters. Bold values with an asterisk denote the selected value of k that maximized the silhouette score.

## Spatial clustering patterns


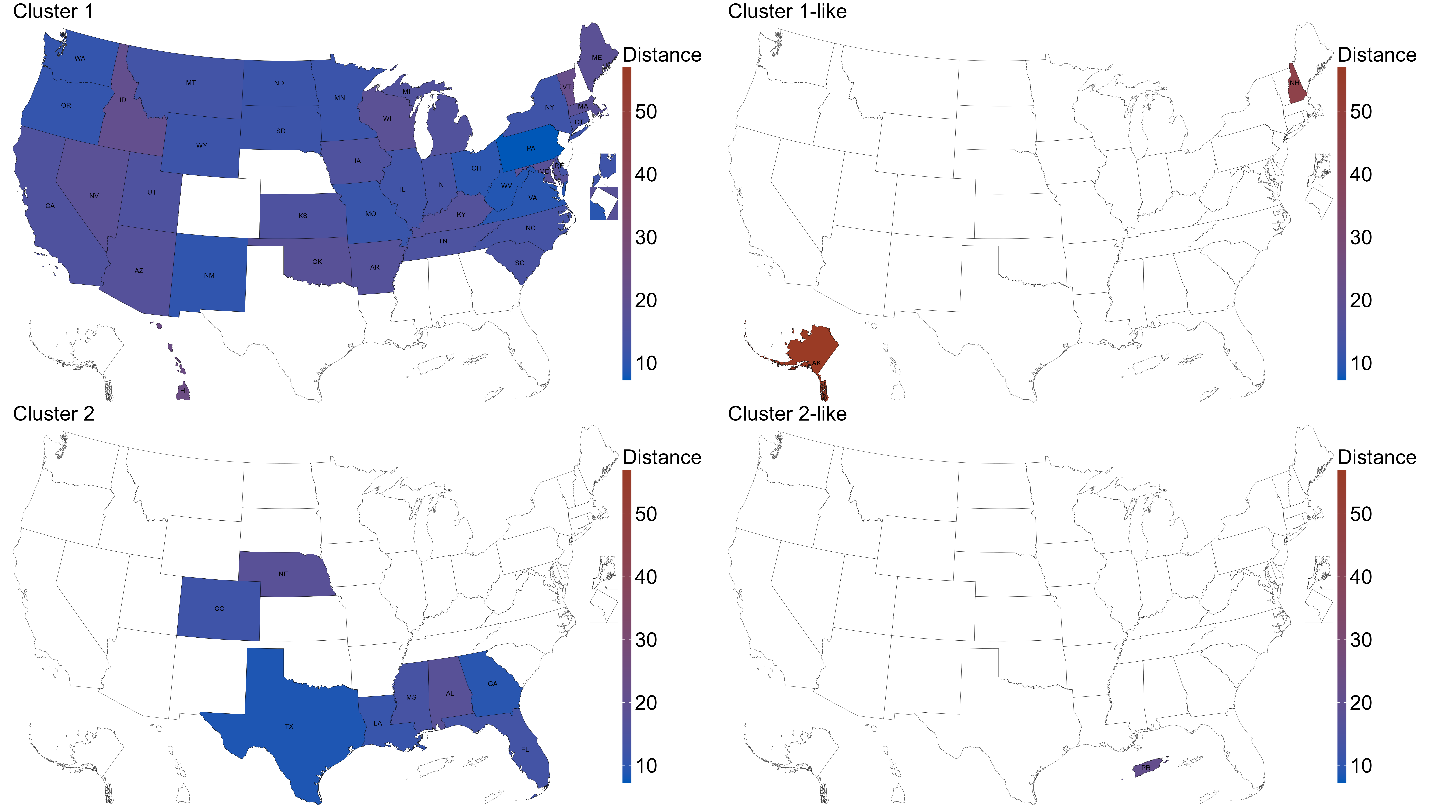


Figure S13. Spatial distribution of cluster distances from their respective centroids, based on the multivariate analysis using both datasets (the unweighted weekly proportions of outpatient visits for ILI and the weekly percentages of specimens testing positive for influenza) for the pre-COVID-19 seasons (2010/2011–2019/2020, upper right panel of Figure 2). The latter consist of both public health and clinical laboratory data reported before the 2015/2016 season, while only the clinical laboratory data were available from the 2015/2016 season onward. The blue color represents shorter distances, while red represents longer distances. The right panels of this figure show outliers, defined as those with a distance greater than one standard deviation from the mean. New Hampshire (NH) and Alaska (AK) are outliers in Cluster 1, while Puerto Rico (PR) is the outlier in Cluster 2. Note that virologic surveillance data from PR were derived from public health laboratory data, which may not reflect diagnostic testing, as both public health and clinical laboratory data were reported together prior to the 2015/2016 season and could not be separated.


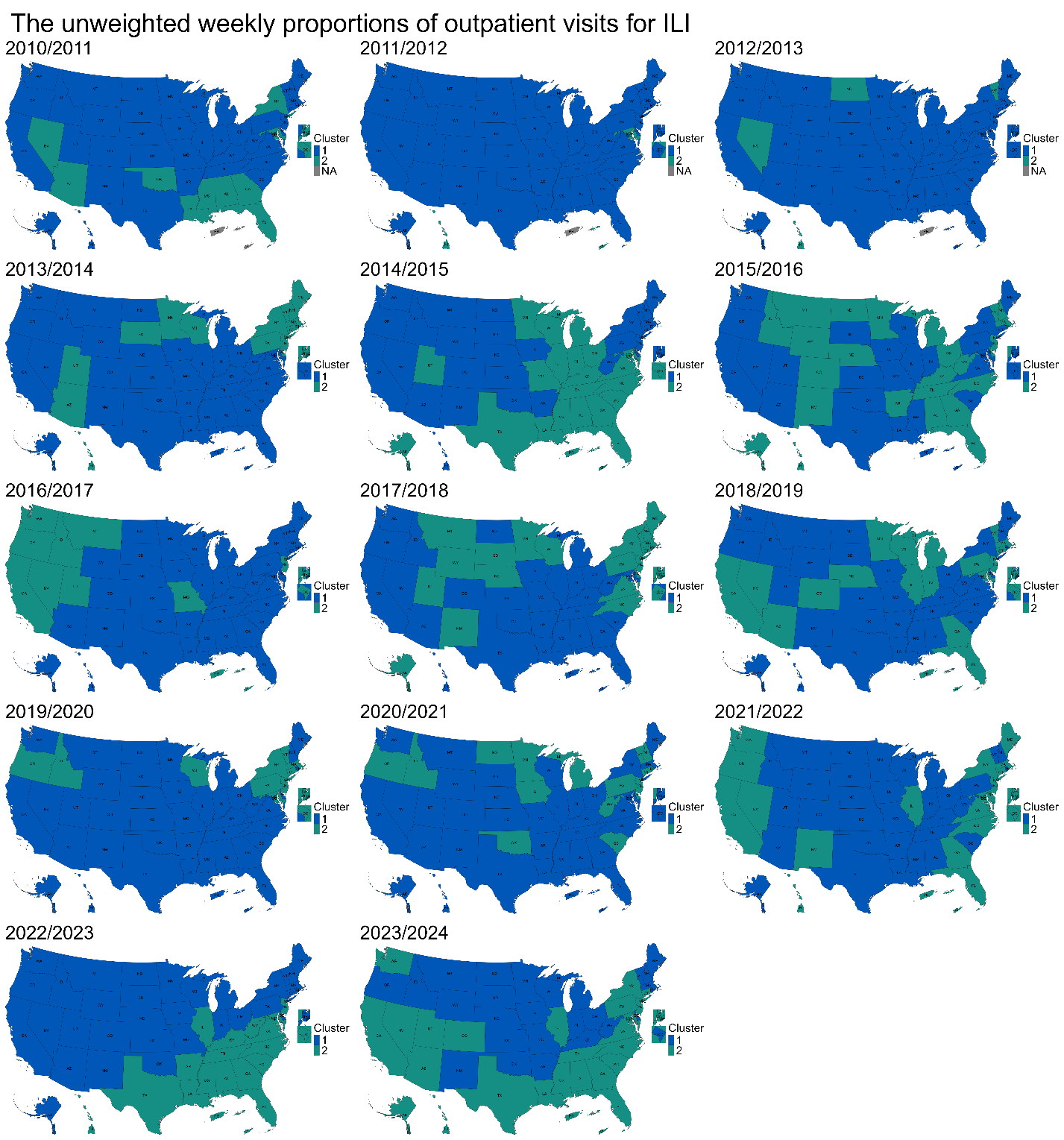


Figure S14. Spatial distribution of clustering patterns from the 2010/2011 to 2023/2024 seasons, based on univariate analysis using the unweighted weekly proportions of outpatient visits for ILI. The blue color represents the largest cluster containing most jurisdictions, while the green color represents the smaller cluster with fewer jurisdictions. The gray jurisdictions were excluded from the analysis due to insufficient data for the respective seasons.


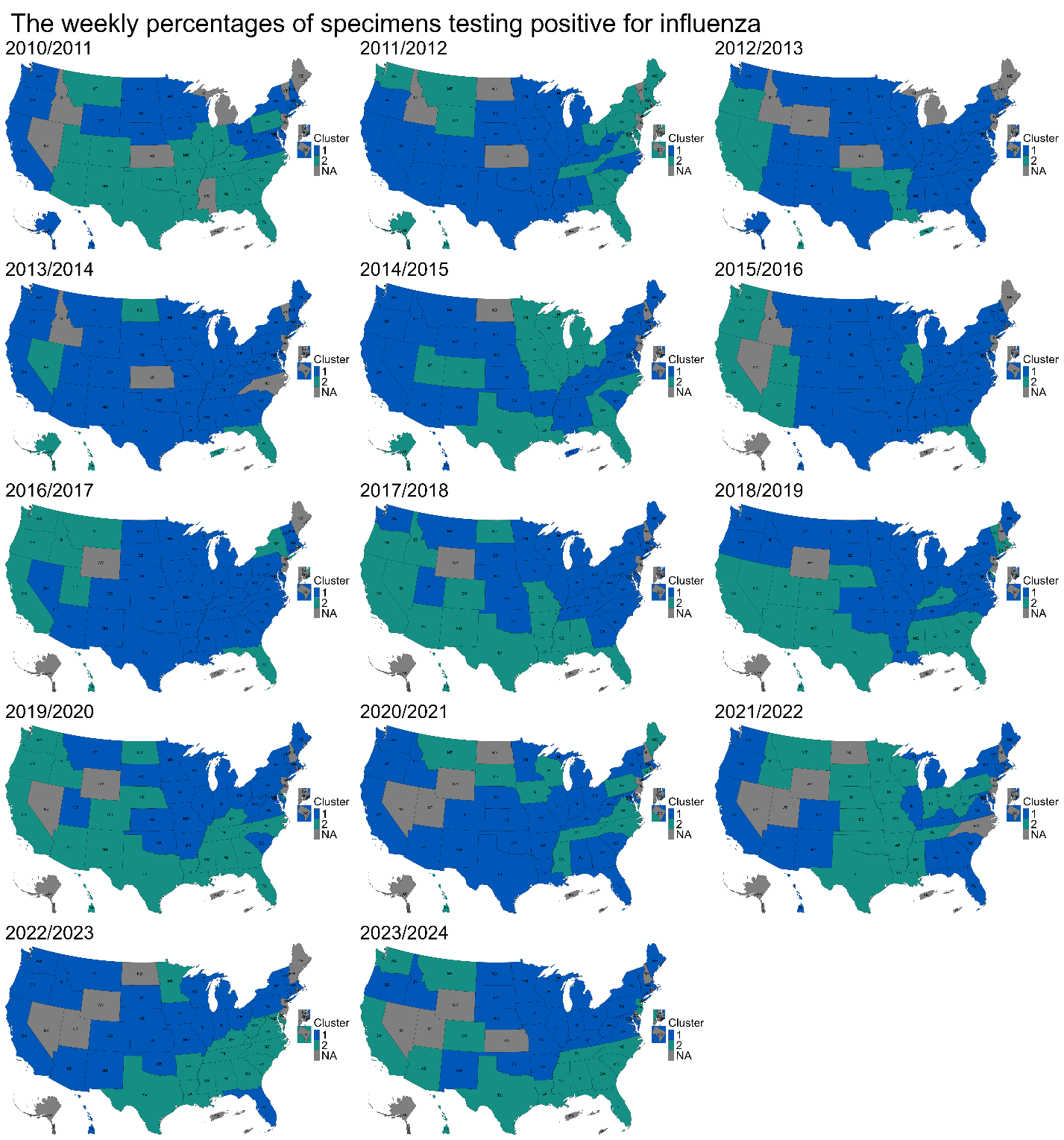


Figure S15. Spatial distribution of clustering patterns from the 2010/2011 to 2023/2024 seasons, based on univariate analysis using the weekly percentages of specimens testing positive for influenza. The data consist of both public health and clinical laboratory data reported before the 2015/2016 season, while only the clinical laboratory data were available from the 2015/2016 season onward. The blue color represents the largest cluster containing most jurisdictions, while the green color represents the smaller cluster with fewer jurisdictions. The gray jurisdictions were excluded from the analysis due to insufficient data for the respective seasons.

## Spatial clustering patterns using shorter bandwidth

As a sensitivity analysis, we repeated the smoothing and subsequent clustering analyses using a shorter bandwidth of $h=1$ week, which closely approximates the unsmoothed weekly series. The resulting clustering patterns were nearly identical to those obtained using the 4-week bandwidth, and the core spatial clustering patterns remained unchanged (Figure S16), indicating that the main findings are robust to the choice of smoothing parameter.


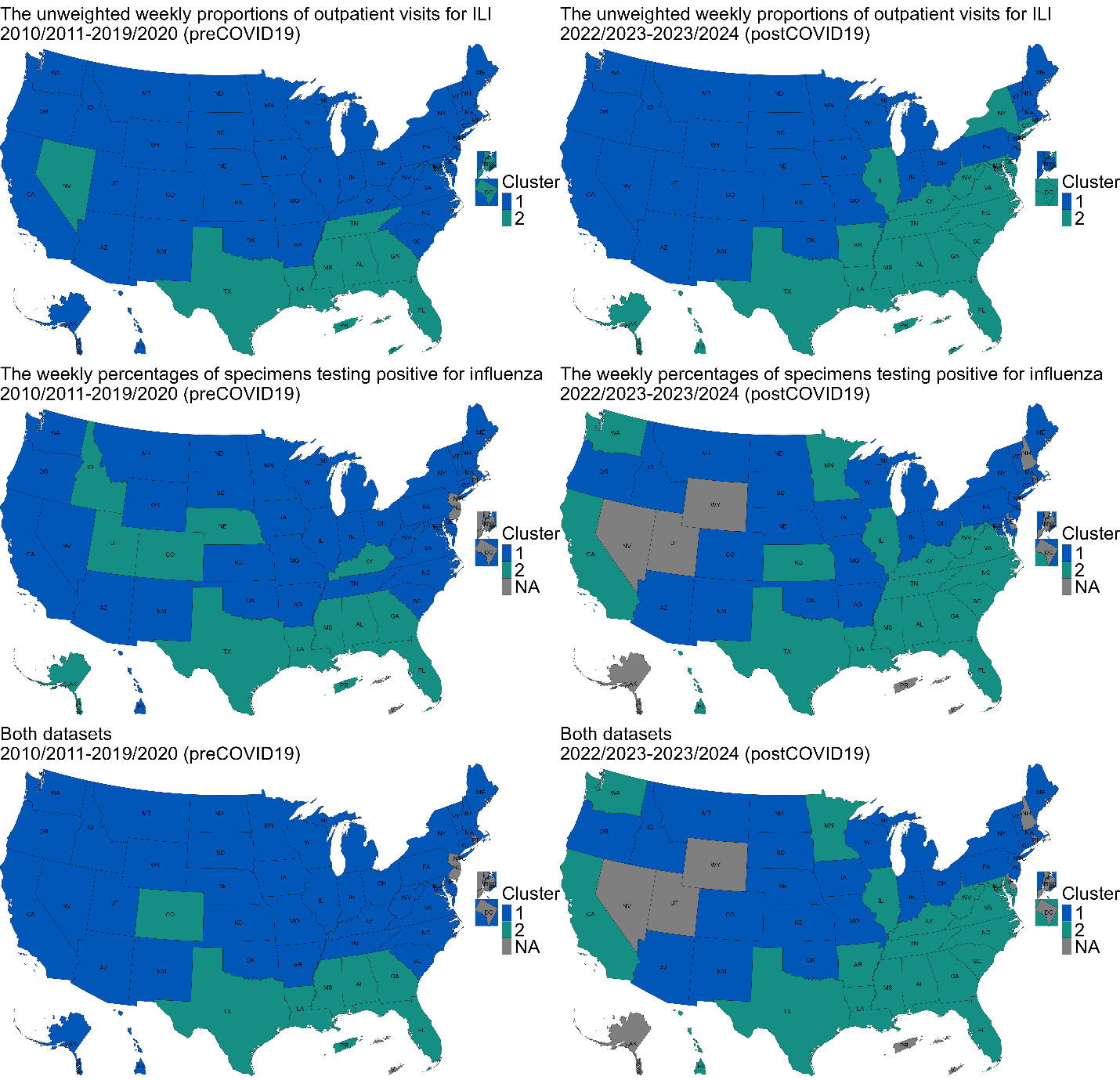


Figure S16. Spatial distribution of clustering patterns for the pre-COVID-19 seasons (2010/2011–2019/2020, left panels) and the post-COVID-19 seasons (2022/2023–2023/2024, right panels). (Upper panels) Univariate analysis based on the unweighted weekly proportions of outpatient visits for ILI, (Middle panels) univariate analysis based on the weekly percentages of specimens testing positive for influenza, and (Lower panels) multivariate analysis using both datasets. The blue color represents the largest cluster containing most jurisdictions, while the green color represents the smaller cluster with fewer jurisdictions. The gray jurisdictions were excluded from the analysis due to insufficient data across all seasons. This figure is identical to Figure 2 except that a 1-week smoothing bandwidth was used instead of the 4-week bandwidth.

## Evaluating cluster differences using ANOVA

We conducted a series of three-way ANOVAs to examine the effects of *CLUSTER* (with values 1, 2, or 3), *SEASON* (10 seasons: 2010/2011–2019/2020), and *DATASET* (the unweighted weekly proportions of outpatient visits for ILI, the weekly percentages of specimens testing positive for influenza, or both) on four key variables: *PEAK_WEEK* (peak timing), *MORAN_I_LOCAL* (local Moran’s I), *PERCENT_A* (proportion of all influenza A and B virus detections that were influenza A viruses), and *PERCENT_H1* (proportion of all influenza A virus detections that were influenza A/H1 viruses). The distributions of each of these variables are shown in Figure S17, Figure S18, Figure S19 and Figure S20. We note that only univariate analyses were performed for *PEAK_WEEK* and *MORAN_I_LOCAL*, and the optimal number of clusters was $k=2$.

The ANOVA summary is provided in Table S2.

- *PEAK_WEEK* (peak timing): Significant differences were found for *SEASON*, *CLUSTER:SEASON*, *CLUSTER:DATASET*, and *CLUSTER:SEASON:DATASET* interactions. This indicates that peak timing varied across seasons, between clusters, and according to the data type, suggesting complex seasonal patterns for influenza.
- *MORAN_I_LOCAL* (local Moran’s I): Significant effects were observed for *CLUSTER*, *SEASON*, *DATASET*, and their interactions (except *CLUSTER:DATASET*). This suggests that local spatial autocorrelation patterns were influenced by clustering, seasonal variations, and data types, with distinct spatial patterns over time.
- *PERCENT_A* (proportion of Influenza A viruses): Significant variation was observed for *SEASON* and the *CLUSTER:SEASON* interaction. This indicates that the distribution of Influenza A varied across seasons and clusters.
- *PERCENT_H1* (proportion of Influenza A/H1 viruses): Significant effects were found for *CLUSTER*, *SEASON*, and the *CLUSTER:SEASON:DATASET* interaction. This shows that the proportion of A/H1 viruses differed between clusters, varied over seasons, and was influenced by the complex interaction of all three factors.


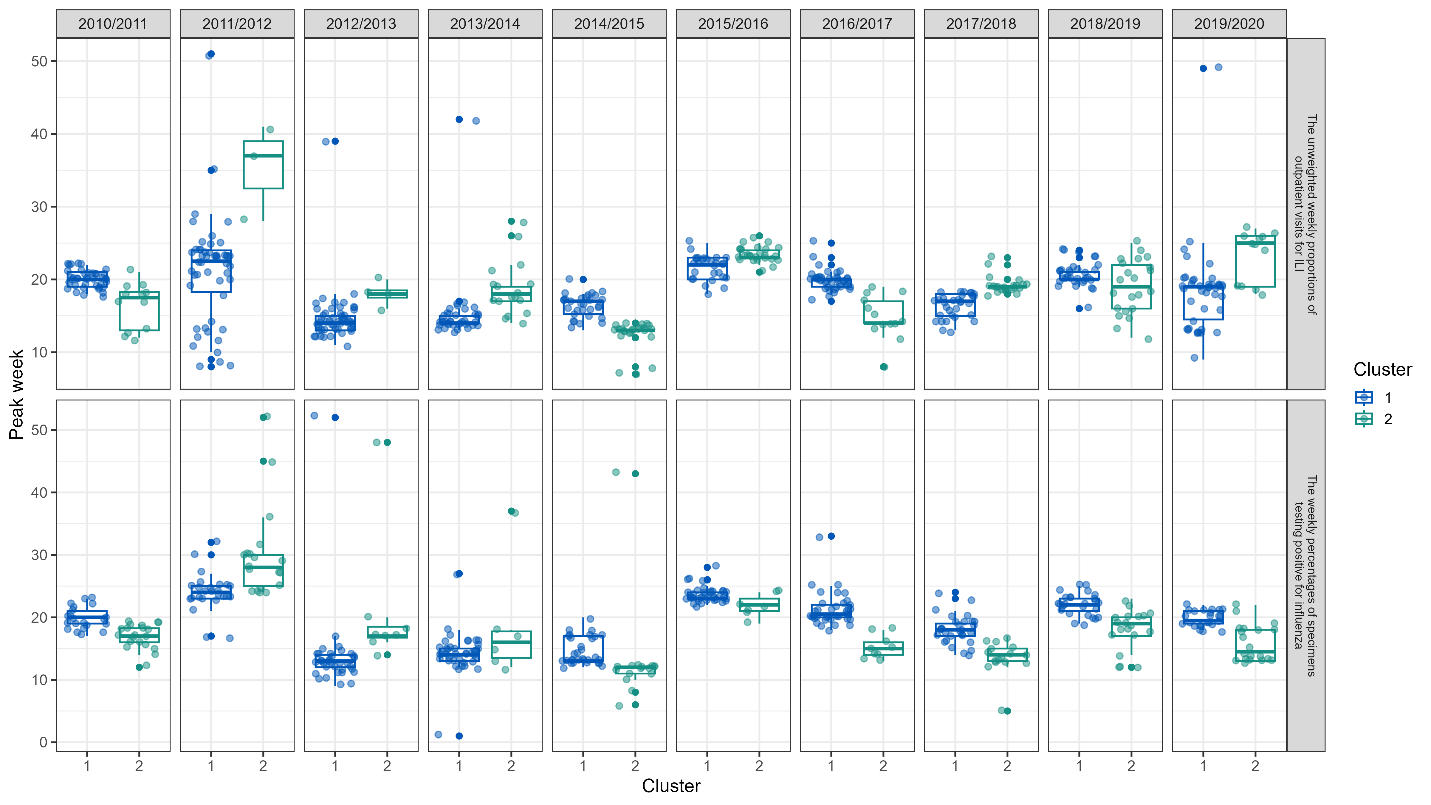


Figure S17. Distributions of peak timing across clusters, seasons and datasets. Only univariate analyses were performed.


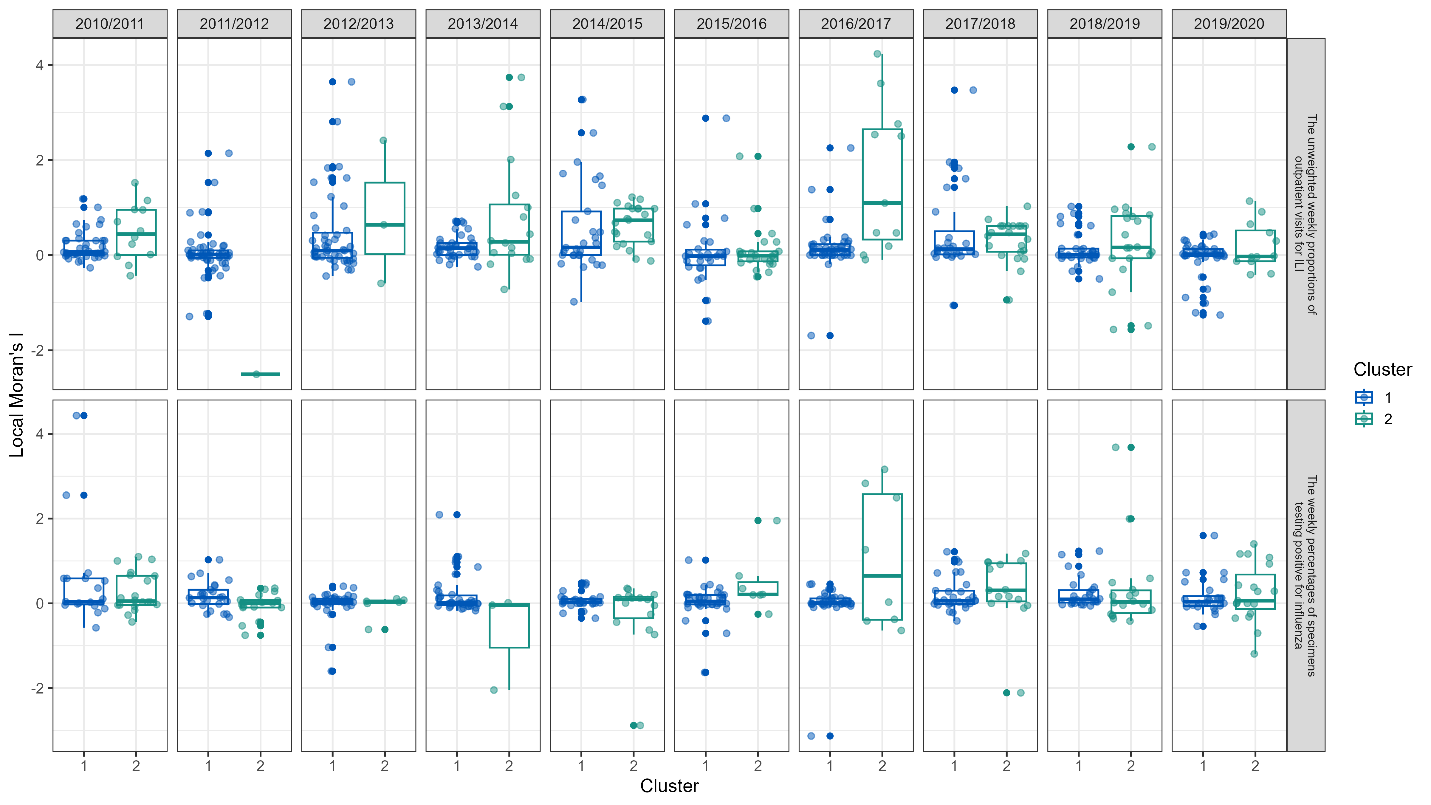


Figure S18. Distributions of local Moran’s I across clusters, seasons and datasets. Only univariate analyses were performed.


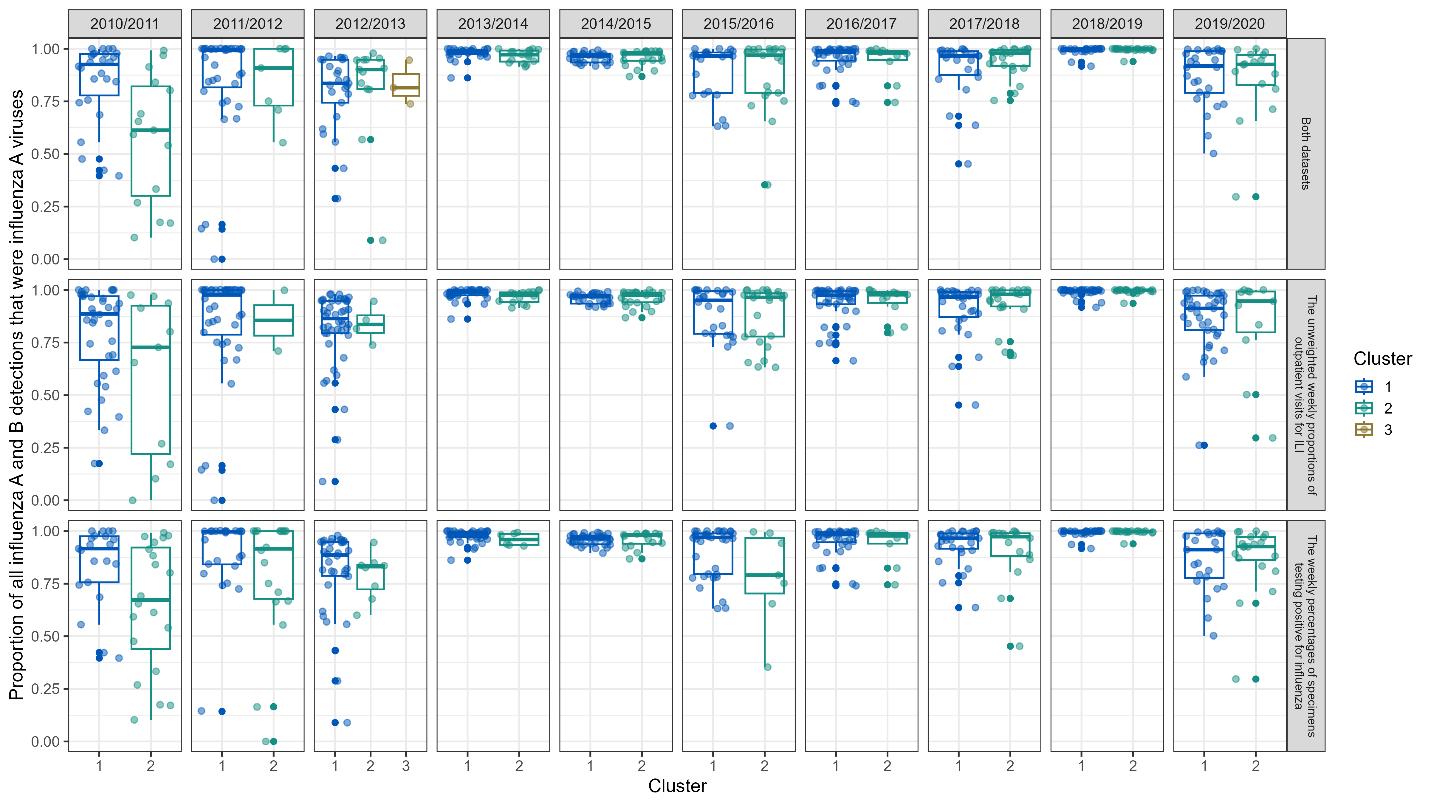


Figure S19. Distributions of the proportion of all influenza A and B virus detections that were influenza A viruses across clusters, seasons and datasets.


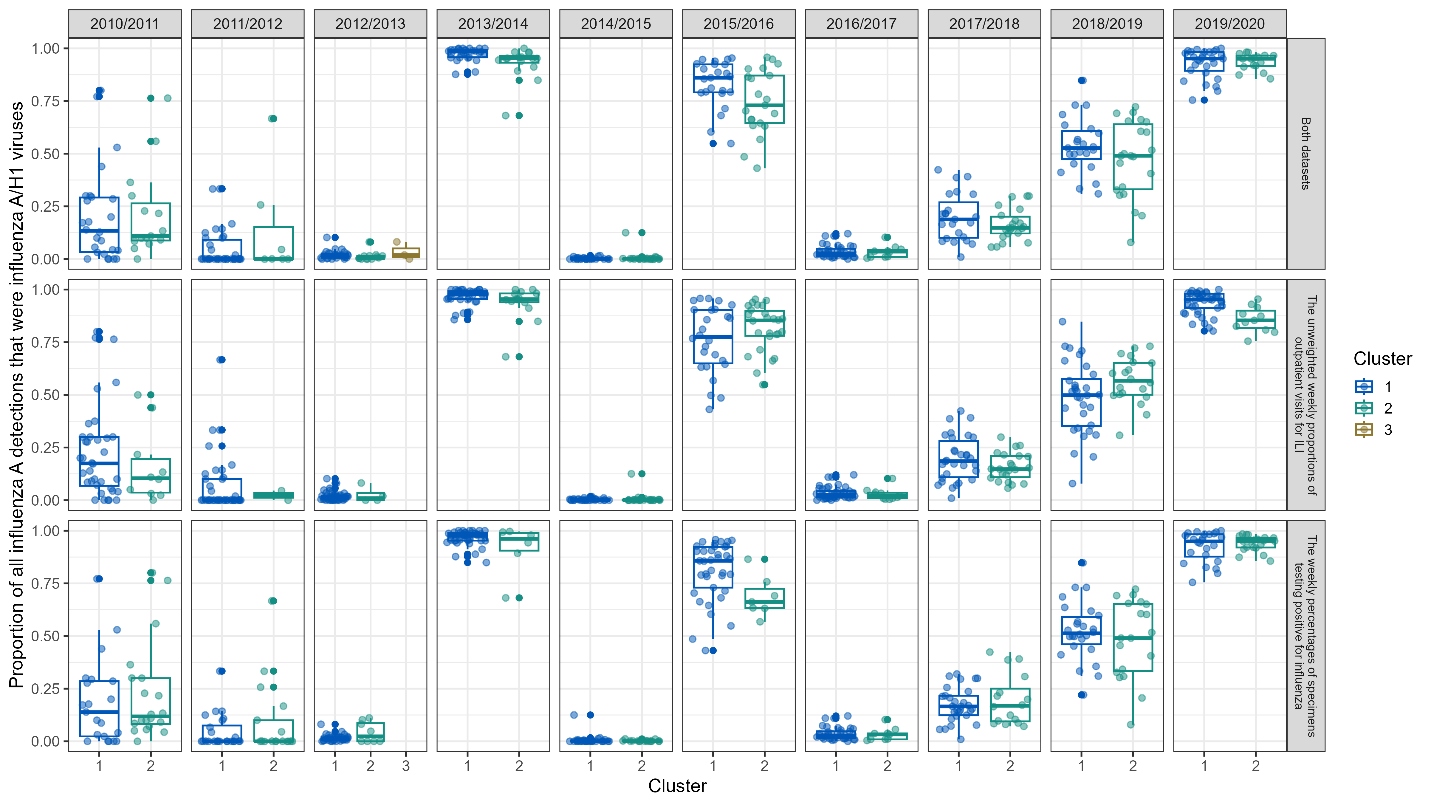


Figure S20. Distributions of the proportion of all influenza A virus detections that were influenza A/H1 viruses across clusters, seasons and datasets.

| **Source of Variation** | **Df** | **Sum Sq** | **Mean Sq** | **F value** | **Pr(>F)** |
| --- | --- | --- | --- | --- | --- |
| ***PEAK_WEEK*** (peak timing) | | | | | |
| *CLUSTER* | 1 | 1 | 0.7 | 0.049 | 0.825 |
| *SEASON* | 9 | 8997 | 999.6 | 67.175 | < 2e-16 *** |
| *DATASET* | 1 | 10 | 10.5 | 0.703 | 0.402 |
| *CLUSTER:SEASON* | 9 | 3007 | 334.1 | 22.453 | < 2e-16 *** |
| *CLUSTER:DATASET* | 1 | 306 | 305.6 | 20.534 | 6.61e-06 *** |
| *SEASON:DATASET* | 9 | 211 | 23.5 | 1.578 | 0.117 |
| *CLUSTER:SEASON:DATASET* | 9 | 758 | 84.2 | 5.661 | 1.18e-07 *** |
| Residuals | 938 | 13958 | 14.9 |  |  |
| ***MORAN_I_LOCAL*** (local Moran’s I) | | | | | |
| *CLUSTER* | 1 | 4.8 | 4.841 | 11.865 | 0.000599 *** |
| *SEASON* | 9 | 10.3 | 1.142 | 2.8 | 0.003036 ** |
| *DATASET* | 1 | 4.1 | 4.06 | 9.951 | 0.001662 ** |
| *CLUSTER:SEASON* | 9 | 24 | 2.669 | 6.542 | 4.58e-09 *** |
| *CLUSTER:DATASET* | 1 | 0.7 | 0.711 | 1.742 | 0.187263 |
| *SEASON:DATASET* | 9 | 17 | 1.886 | 4.622 | 5.41e-06 *** |
| *CLUSTER:SEASON:DATASET* | 9 | 13 | 1.441 | 3.531 | 0.000258 *** |
| Residuals | 884 | 360.7 | 0.408 |  |  |
| ***PERCENT_A*** (proportion of all influenza A and B virus detections that were influenza A viruses) | | | | | |
| *CLUSTER* | 2 | 0.12 | 0.0601 | 2.781 | 0.0623 |
| *SEASON* | 9 | 7.667 | 0.8519 | 39.434 | < 2e-16 *** |
| *DATASET* | 2 | 0.003 | 0.0015 | 0.068 | 0.9341 |
| *CLUSTER:SEASON* | 9 | 1.192 | 0.1324 | 6.128 | 1.75e-08 *** |
| *CLUSTER:DATASET* | 2 | 0.021 | 0.0106 | 0.493 | 0.6111 |
| *SEASON:DATASET* | 18 | 0.091 | 0.005 | 0.233 | 0.9997 |
| *CLUSTER:SEASON:DATASET* | 18 | 0.162 | 0.009 | 0.416 | 0.9851 |
| Residuals | 1314 | 28.386 | 0.0216 |  |  |
| ***PERCENT_H1*** (proportion of all influenza A virus detections that were influenza A/H1 viruses) | | | | | |
| *CLUSTER* | 2 | 0.48 | 0.242 | 20.221 | 2.24e-09 *** |
| *SEASON* | 9 | 189.66 | 21.074 | 1758.289 | < 2e-16 *** |
| *DATASET* | 2 | 0 | 0 | 0.001 | 0.999 |
| *CLUSTER:SEASON* | 9 | 0.11 | 0.013 | 1.061 | 0.3893 |
| *CLUSTER:DATASET* | 2 | 0.02 | 0.008 | 0.694 | 0.4999 |
| *SEASON:DATASET* | 18 | 0.02 | 0.001 | 0.095 | 1 |
| *CLUSTER:SEASON:DATASET* | 18 | 0.47 | 0.026 | 2.159 | 0.0033 ** |
| Residuals | 1310 | 15.7 | 0.012 |  |  |

Table S2. Results of three-way ANOVAs examining the effects of CLUSTER (with values 1, 2, or 3), SEASON (10 seasons: 2010/2011–2019/2020), and DATASET (the unweighted weekly proportions of outpatient visits for ILI, the weekly percentages of specimens testing positive for influenza, or both) on four key variables: PEAK_WEEK (peak timing), MORAN_I_LOCAL (local Moran’s I), PERCENT_A (proportion of all influenza A and B virus detections that were influenza A viruses), and PERCENT_H1 (proportion of all influenza A virus detections that were influenza A/H1 viruses). Each column presents degrees of freedom (Df), sum of squares (Sum Sq), mean squares (Mean Sq), F-values, and p-values (Pr(>F)) for main effects and interactions. Significance levels are indicated as: *** p < 0.001, ** p < 0.01, * p < 0.05. Observations deleted due to missingness were 642 (PEAK_WEEK), 696 (MORAN_I_LOCAL), 245 (PERCENT_A), and 249 (PERCENT_H1).
